# NEDD4 lactylation promotes APAP induced liver injury through Caspase11 dependent non-canonical pyroptosis

**DOI:** 10.7150/ijbs.91284

**Published:** 2024-02-04

**Authors:** Qinglin Li, Fengping Zhang, Hai Wang, Yingmu Tong, Yunong Fu, Kunjin Wu, Jing Li, Cong Wang, Zi Wang, Yifan Jia, Rui Chen, Yang Wu, Ruixia Cui, Yi Wu, Yun Qi, Kai Qu, Chang Liu, Jingyao Zhang

**Affiliations:** 1 Department of Hepatobiliary Surgery, The First Affiliated Hospital of Xi'an Jiaotong University, Xi'an Shaanxi 710061, People's Republic of China.; 2 Key Laboratory of Surgical Critical Care and Life Support (Xi'an Jiaotong University), Ministry of Education, People's Republic of China.; 3 Department of Vascular Surgery, Xiangya Hospital, Central South University, Changsha, People's Republic of China.; 4 Department of Hepatobiliary Surgery and Liver Transplantation, The Second Affiliated Hospital of Xi'an Jiaotong University, People's Republic of China.; 5 Department of SICU, The First Affiliated Hospital of Xi'an Jiaotong University, Xi'an Shaanxi 710061, People's Republic of China.; 6 Department of General Surgery, The First Affiliated Hospital of Xi'an Jiaotong University, Xi'an Shaanxi 710061, People's Republic of China.; 7 Department of Vascular Surgery, The First Affiliated Hospital of Xi'an Jiaotong University, Xi'an Shaanxi 710061, People's Republic of China.; 8 MOE Key Laboratory of Environment and Genes Related to Diseases, School of Basic Medical Sciences, Xi'an Jiaotong University, Xi'an Shaanxi 710061, People's Republic of China.; 9 Department of Ophthalmology, The First Affiliated Hospital of Xi'an Jiaotong University, Xi'an Shaanxi 710061, People's Republic of China.

**Keywords:** caspase-11, non-canonical pyroptosis, lactate, lactylation, NEDD4.

## Abstract

Caspase-11 detection of intracellular lipopolysaccharide mediates non-canonical pyroptosis, which could result in inflammatory damage and organ lesions in various diseases such as sepsis. Our research found that lactate from the microenvironment of acetaminophen-induced acute liver injury increased Caspase-11 levels, enhanced gasdermin D activation and accelerated macrophage pyroptosis, which lead to exacerbation of liver injury. Further experiments unveiled that lactate inhibits Caspase-11 ubiquitination by reducing its binding to NEDD4, a negative regulator of Caspase-11. We also identified that lactates regulated NEDD4 K33 lactylation, which inhibits protein interactions between Caspase-11 and NEDD4. Moreover, restraining lactylation reduces non-canonical pyroptosis in macrophages and ameliorates liver injury. Our work links lactate to the exquisite regulation of the non-canonical inflammasome, and provides a basis for targeting lactylation signaling to combat Caspase-11-mediated non-canonical pyroptosis and acetaminophen-induced liver injury.

## Introduction

Non-canonical pyroptosis is a programmed cell death mediated by Caspase-11 and GSDMD^1^, ^2^. It is initiated after activation of Caspase-11 after discriminating LPS in the cell. Then, the activated Caspase-11 cleaves the GSDMD, leading to the release of N-GSDMD with membrane pore-forming activity^3^, ^4^. The process of non-canonical pyroptosis was first revealed in immune cells such as macrophages and has a marked effect on a variety of inflammatory diseases such as sepsis, which is often accompanied by the aggravation and spread of inflammation^5^-^7^. Therefore, how to regulate non-canonical pyroptosis is essential for mitigating inflammation-induced injury. Recent studies suggested that metabolites and metabolites-mediated post-translational modifications of proteins can impact the canonical inflammasome and cell pyroptotic pathway. However, whether metabolites affect the non-canonical pyroptosis remains unclear^8^, ^9^.

Lactate is one of the most important metabolites. Meanwhile, lactate has been proven to be a prognostic marker for many diseases. Patients with high-level lactate, particularly in infectious diseases such as sepsis, have a significantly worse prognosis^10^. Recent research has revealed that lactate regulates the function of immune cells, acts as a messenger molecule to influence cell signaling pathways, and potentially affect the process of cell death^11^, ^12^. Simultaneously, Zhang et al. found that lactate can promote lactylation, a new post-translational modification that can occur on a variety of proteins^13^, ^14^. Studies have shown that lactylation of histones can regulate gene transcription, while lactylation of non-histone proteins may affect localization and function^15^-^17^. Lactylation also plays a vital role in inflammation and regeneration, but it is not yet clear whether lactate and its promotion of lactylation regulate cell pyroptosis through specific protein functions^14^, ^15^.

The ubiquitination of inflammasome proteins is one of the most critical mechanisms that regulate pyroptosis and inflammatory responses^18^. In non-canonical pyroptosis, NEDD4 has been shown to negatively regulate the level of Caspase-11 through ubiquitination, thus inhibiting non-canonical pyroptosis^19^, ^20^. As a classical E3 ubiquitin ligase, NEDD4's function is significantly regulated by post-translational modifications, and some studies suggest that the glycolytic process is related to NEDD4's function^21^, ^22^. Therefore, it is urgently necessary to clarify whether lactate, the most important product of glycolysis, can lactylate NEDD4 and regulate its function.

APAP-induced liver injury (AILI), an acute liver injury resulting from drug overdoses^23^.This disease is also the leading cause of liver transplantation in the US^24^. It has been proven that oxidative stress dominated the primary injury of AILI, but it is unclear whether other factors affect the secondary damage of this process. Evidence has shown that lactate is an adverse prognostic factor for AILI^25^. However, whether the molecular mechanism of lactate can play a causal role in AILI is still unknown. LPS, one of the most important pathogenic substances produced by Gram-negative bacteria, was shown to play a role in AILI^26^-^31^. But it is currently unclear whether non-canonical pyroptosis induced by LPS activation also contributes to the AILI process.

Here, we aimed to explore the role of lactate and lactylation in Caspase-11 mediated non-canonical pyroptosis. We found that lactate promotes this process through a NEDD4-lactylation-mediated pathway. Our result also indicated that non-canonical pyroptosis promotes AILI.

## Materials and Methods

### Cell lines, reagents, and animals

L929, HEK293, HEK293T cell lines were obtained from the ATCC. Cell lines were cultured in DMEM with 10% FBS (Gibco). iBMDM cells were a gift from Professor Feng Shao (NIBS). BMDMs, derived from C57BL/6 mice, were isolated and subsequently cultured in DMEM supplemented with 10% FBS and 15% L929 supernatant, known as the BMDM culture medium. The supernatants from L929 cells were gathered and subsequently subjected to centrifugation at a speed of 4000 revolutions per minute for a duration of 10 minutes. Following that, the liquid above the sediment was passed through filters with a pore size of 0.22 μM in order to eliminate any particles and then kept at a temperature of -80 °C. Mouse primary hepatocytes were isolated as described^32^. To determine the level of Caspase-11 expression, BMDM and iBMDM cells were treated with 500 ng/ml LPS for the specified durations.The method for LPS transfection followed a previously described approach^33^. After transfection, the conditioned media (CM) from LPS-transfected BMDMs were collected and centrifuged to eliminate cell debris. Subsequently, the CM was combined with hepatocytes at a ratio of 1 volume of CM to 1 volume of DMEM supplemented with 10% fetal bovine serum. The cells were cultured in a humid incubator with 5% CO_2_ at a temperature of 37°C. Tables S1 provide a comprehensive description of the reagents and antibodies utilized in this investigation.

Approval for this study was granted by the Laboratory Animal Care Committee of Xi'an Jiaotong University. C57BL/6 mice were acquired from Xi'an Jiaotong University's Laboratory Animal Center.*Casp11*^-/-^ mice were acquired from Cyagen Biosciences Inc. Both wild type and gene knock-out mice originate from identical genetic backgrounds. Professional staff followed the “Animal Research Reporting of In Vivo Experiments” (ARRIVE) guidelines and the National Institutes of Health's Guide for the Care and Use of Laboratory Animals to perform all animal care and handling procedures. The animals were kept in conditions that were free of specific pathogens (SPF) and followed a 12-hour light-dark cycle. The display of all the endeavor to reduce the quantity of animals utilized and the agony experienced by animals was evident. We ensured that the temperature and humidity were appropriate, and we made sure there was an abundant supply of food and water. Briefly, mice aged 6-8 weeks were subjected to a 12-hour fasting period prior to intraperitoneal injection of either 400 or 1000 mg/kg APAP dissolved in saline. During the treatment experiments, C646 was administered intraperitoneally to either WT or KO mice 30 minutes after the injection of APAP.

Data from clinical research were acquired from XJTUMC, the Medical College of Xi'an Jiaotong University. The data from XJTUMC were acquired from the Biobank of the Center. The Institutional Review Board of the First affiliated hospital of Xi'an Jiaotong University reviewed this study and exempted it from the need for ethics approval.Since all individuals in this dataset were anonymized, the requirement for informed consent was waived. From August 2011 to June 2022, individuals in the First affiliated hospital of Xi'an Jiaotong University were diagnosed based on the following criteria: (1) presence of liver damage symptoms, (2) recent medication usage, and (3) absence of other liver ailments.

### Transwell co-culture system

To conduct transwell co-culture experiments, cells were placed in 6-well cultured plates containing transwell chamber inserts (Falcon) with a pore size of 0.4 μm.Hepatocytes were placed in the upper compartments and exposed to APAP (10 mM) for the specified duration.Afterwards, the liver cells were co-incubated with BMDMs that were placed in the lower compartments for a duration of 6 hours.

### Plasmids and transfection

The description of pcDNA3.1(-)-HA-Nedd4 has been provided before^34^. pCS2-caspase-11 were presents from Professor Feng Shao (NIBS) and have been previously explained^3^. Lipofectamine 3000 was used to transfect HEK293 and iBMDM cells with the specified expression plasmids. Subsequent experiments necessitated the use of various lysis buffers to lyse the cells. Shanghai Genechem Co.,Ltd. constructed and supplied the shRNA adenovirus related vectors for NEDD4, SIRT1, and P300.

### Cell counting kit-8 (CCK8) assays

The cells were placed in well plates and allowed to grow overnight. Once the cells attached to the surface, they received the designated procedure. Following the treatment, CCK-8 solution was introduced to every well in a 1:10 proportion, and the cells were then incubated for an additional 4 hours. The absorbance of the plates was measured at a wavelength of 450 nm for each group.

### Cell death kinetics

The previous literature reports were used to conduct cell death kinetics assays.^35^.

### Cytotoxicity assay

The measurement of cytotoxicity was conducted using a Cytotoxicity Detection Kit (LDH) as per the instructions provided by the manufacturer. Each independent experiment involved performing assays in triplicate.

### Enzyme linked immunosorbent assay (ELISA)

ELISA kits (Invitrogen) were used to detect murine cytokines, following the manufacturer's protocols.

### Endotoxin Measurements

Plasma endotoxin levels were assessed by employing the QCL Chromogenic LAL Assay (Biowhittaker, Walkersville, MD) in accordance with the guidelines provided by the manufacturer. In order to reduce product inhibition, the samples were diluted by a factor of 1:10 and then subjected to heating at a temperature of 70°C for a duration of 10 minutes before the assay. The assay was conducted using containers and water that were free from endotoxins.

### Lactate measurements

The serum was obtained by spinning clotted whole blood at a speed of 2500 rpm for a duration of 10 minutes. A volume of 1 liter serum was mixed with 49 liters of Lactate Assay Buffer (Sigma) to measure lactate levels. The diluted serum samples (50 μL) were combined with a 50 μL reaction mixture as per the guidelines provided by the manufacturer (Sigma). After incubating the reaction for 30 minutes at ambient temperature, it was measured at 450 nanometers (A450). In order to assess the levels of lactate inside the cells, the cells were rinsed with chilled PBS and then homogenized in approximately 150 μL of the Lactate Assay Buffer, using a pipette to mix the solution by repeatedly moving it up and down. The centrifugation at a speed of 14,000 rpm for 5 minutes effectively eliminated the material that could not be dissolved. The liquid above the sediment was gathered in order to quantify the amount of lactate, following the guidelines provided by the manufacturer. Total cell number was used to normalize intracellular lactate levels.

### Histological analyses

The liver tissues were preserved in 4% paraformaldehyde, encased in paraffin, cut into sections that were 5 μm thick, and subsequently treated with hematoxylin-and-eosin (HE) stain, following standard protocols. The histological scores were determined by adding up the scores for each of the six items, which included cytoplasmic color fading, vacuolization, nuclear condensation, nuclear fragmentation, nuclear fading, and erythrocyte stasis. The score grades ranged from 0 (indicating no change) to 3 (indicating severe changes) for each item. The overall score varied between 0 and 18^36^. Immunohistochemical analyses were facilitated by preparing sections through deparaffinization, and then retrieving antigens by boiling them in the citrate buffer. Next, the sections were treated with 0.3% Triton X-100 to make them permeable. To prevent non-specific binding, 1% bovine serum albumin (BSA) was employed, followed by overnight incubation with primary antibodies at 4°C. Following the PBS wash, the specimens were exposed to a biotin-conjugated secondary antibody and streptavidin HRP-complex for 1 hour at ambient temperature. DAB (3,3'-diaminobenzidine) was utilized to generate the signals, followed by counterstaining the sections with hematoxylin. Macrophages per treatment were seeded onto confocal dishes and fixed using 4% paraformaldehyde for immunofluorescent analyses. The tissue samples were placed in the ideal freezing temperature (OCT) substance and sliced into 20-μM-thick pieces using a cryostat. To permeabilize the cells on coverslips and tissue sections, 0.3% Triton X-100 was used, followed by blocking with 1% BSA at room temperature. Subsequently, the samples were incubated with primary antibodies overnight at 4 °C. Following PBS washing, the specimens were exposed to a secondary antibody and DAPI was used to stain the cell nuclei for 15 minutes at ambient temperature. In the end, the cells and tissues that had been stained were examined and photographed using either a Leica Microsystems CMS GmbH light microscope, a Zeiss Axio Vert. A1fluorescent microscope, or a Leica Microsystems confocal microscope.

### Reverse transcription-quantitative polymerase chain reaction (RT-qPCR)

Cells were subjected to RNA extraction using Trizol reagent, followed by reverse transcription into cDNA using the PrimeScrip™ RT reagent kit. The SYBR green premix kit and ABI 7900HT Fast Reverse transcription-PCR system were utilized for qPCR. The 2^-ΔΔCT^ method was utilized to calculate the relative mRNA levels. To standardize the gene expression levels, GAPDH was employed as an internal reference. Table S2 contains the listed primer sequences.

### Immunoblotting

RIPA lysis buffer, along with a protease inhibitor cocktail, was used to lyse cells or tissue samples. A NanoDrop spectrophotometer was utilized to measure the protein concentrations of the lysates. Equal amounts of protein lysates were analyzed by SDS-PAGE, and the resulting protein bands were then transferred onto PVDF membranes. The membranes were obstructed with a 5% BSA solution and subsequently exposed to primary antibodies overnight at a temperature of 4 °C. After washing, the membranes were exposed to a secondary antibody conjugated with HRP for 1 hour at ambient temperature. In the end, the protein bands of interest were detected by employing an enhanced chemiluminescence (ECL) plus reagent. The analysis of images was conducted using ImageJ software (NIH, USA), version 1.53.

### In vivo ubiquitination assays

After 24 hours of transfection, MG132 (10 µM) was introduced for 8 hours. Following this, the cells were lysed using a strong RIPA lysis buffer that included protease inhibitors (1:500) and 1% SDS. The lysing process was carried out on ice for 1 hour, and subsequently, the cells were denatured by heating for 5 minutes. Following three rounds of ultrasonication, the supernatants were diluted using lysis buffer until the SDS concentration reached 0.1%, and subsequently subjected to re-immunoprecipitation using the suitable antibodies. Lysates were immunoprecipitated using an anti-Flag antibody for an extended period at a temperature of 4 °C, and subsequently pulled down using protein A/G beads. After being washed thrice with RIPA lysis buffer, the samples were separated by gel and subjected to analysis through immunoblotting using anti-Ubi antibody.

### In vitro ubiquitination assays

In the vitro ubiquitination test, Casp-11 that had been purified was mixed with HA-NEDD4, HA-NEDD4K33R, and P300 that had been immunoprecipitated from cell lysates. This mixture was then combined with purified ubiquitin, E1, E2, Mg^2+^-ATP, and DTT (50 mM) in a total volume of 50 μL for the reaction. The contents were gently combined and placed in an incubator at a temperature of 37°C for a duration of 1 hour. To stop the reaction, 50μL of 2x SDS loading buffer was added, followed by heating to either 95°C for 5 minutes or 70°C for 10 minutes. The ubiquitination of Casp11 was examined using immunoblotting and the anti-Ubi antibody.

### Co-immunoprecipitation

To perform immunoprecipitation, the whole-cell extracts were lysed using IP lysis buffer (Beyotime) containing a protease inhibitor cocktail (Millipore) and PMSF (Beyotime). If needed, a 1% solution of SDS was employed to interrupt the interaction between proteins at a temperature of 90°C for a duration of 5 minutes. After centrifuging cell lysates at 12,000×g for 15 minutes, the resulting supernatants were collected and then mixed with protein A/G magnetic beads (MedChemExpress) that had been pre-bound with specific antibodies. Following an overnight incubation, the magnetic beads underwent five washes with IP wash buffer and were then eluted using SDS-PAGE loading buffer for subsequent detection.

### LC-MS for lactylation detection

After transfecting HEK293 cells with the HA-NEDD4 plasmid for 24 hours, they were subsequently exposed to L-lactic acid for the same duration. Following this, the cell lysates underwent treatment with 1% SDS and were then subjected to immunoprecipitation using an antibody specific to the HA epitope tag. Based on the data given by PTM Inc. The procedures used in the experiment were as follows.

#### In-gel Digestion

To perform in-gel tryptic digestion, the gel pieces were destained using a solution of 50 mM NH_4_HCO_3_ in 50% acetonitrile (v/v) until they became transparent. The gel pieces were dried using 100 μl of pure acetonitrile for 5 minutes, the liquid was then removed, and the gel pieces were soaked in 10 mM dithiothreitol and kept at 37 °C for 60 minutes. Gel pieces were again dehydrated in 100% acetonitrile, liquid was removed and gel pieces were rehydrated with 55 mM iodoacetamide. The samples were kept in a dark environment at ambient temperature for a duration of 45 minutes. The gel pieces were rinsed using a solution of 50 millimolar NH_4_HCO_3_ and then dried using pure acetonitrile. The gel pieces were hydrated with trypsin resuspended in 50 mM NH_4_HCO_3_ at a concentration of 10 ng/μl and kept on ice for 1 hour. After removing the excess liquid, the gel pieces were then subjected to trypsin digestion at 37 °C for the entire night. The peptides were obtained by using a mixture of 50% acetonitrile and 5% formic acid, and then with pure acetonitrile. The peptides were completely dried and then reconstituted in a solution containing 2% acetonitrile and 0.1% formic acid.

#### LC-MS/MS Analysis

The tryptic peptides were dissolved in 0.1% formic acid and 2% acetonitrile (solvent A), directly loaded onto a home-made reversed-phase analytical column (15-cm length, 75 μm i.d.). The slope consisted of a rise from 6% to 25% solvent B (0.1% formic acid in 90% acetonitrile) within a duration of 16 minutes, followed by an increase from 25% to 35% in 6 minutes. Subsequently, it rapidly reached 80% in 4 minutes and remained steady at 80% for the final 4 minutes. These changes occurred while maintaining a consistent flow rate of 450 nl/min using an EASY-nLC 1200 UPLC system.

The peptides underwent NSI source and were then analyzed using tandem mass spectrometry (MS/MS) in Q ExactiveTM Plus (Thermo) connected online to the UPLC. A voltage of 2.1 kilovolts was applied for electrospray. The range of m/z scan was from 350 to 1800 for full scanning, and intact peptides were identified in the Orbitrap with a resolution of 60,000. Following the selection of peptides, MS/MS was performed using an NCE setting of 28, and the fragments were subsequently detected in the Orbitrap with a resolution of 15,000. The procedure relied on data and involved alternating between a single MS scan and 20 MS/MS scans, each followed by a 15.0s dynamic exclusion. The automatic gain control (AGC) was adjusted to 50,000.

#### Data Processing

PEAKS Studio 10.6 build 20201221 was utilized to process the obtained MS/MS data. The target protein sequence was used to search the tandem mass spectra against the database. Up to 2 missing cleavages were allowed, specifying Trypsin/P (or other enzymes if available) as the cleavage enzyme. The mass error for precursor ions was set to 10 parts per million (ppm), while for fragment ions it was set to 0.02 Daltons (Da). Fixed modification was specified for carbamidomethyl on Cys, while variable modification was specified for oxidation on Met. The confidence level for peptides was set to high, with a peptide ion score greater than 15.

### Statistical analysis

The information was examined utilizing SPSS 22.0 program and displayed as average ± standard deviation. Student's t-test was used to calculate the differences between two groups, while one-way analysis of variance (ANOVA) was used to calculate the differences among more than two groups. The notation 'n=3' indicates that it represents a minimum of three separate experiments. We utilized the Kaplan-Meier methodology to plot survival curves and subsequently analyzed them using the log-rank test. To correlate the expression levels of various genes, the Spearman's rank correlation test was employed. Significant differences were deemed for P-values less than 0.05.

## Results

### Knock-out of Caspase-11 ameliorate APAP-induced liver injury in mice

**Figure 1:**
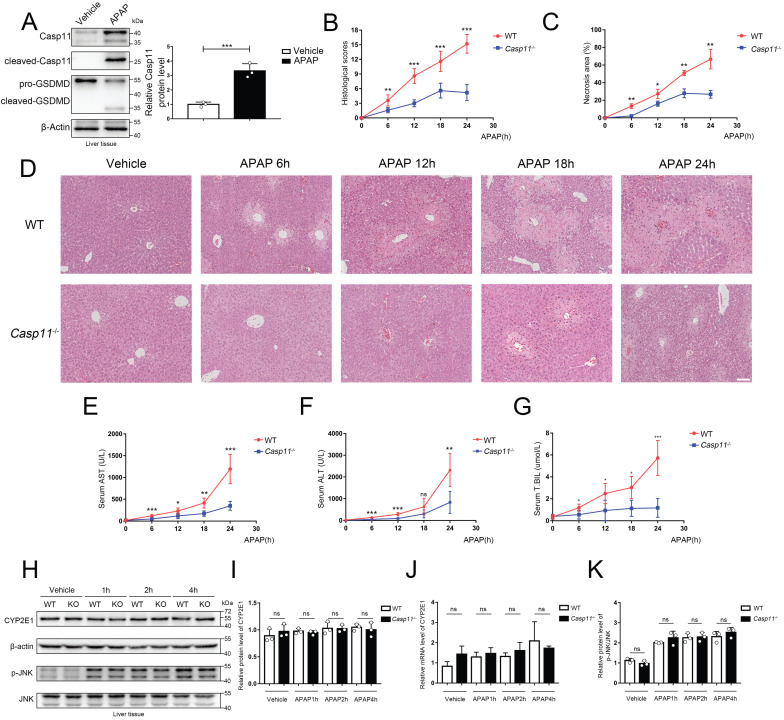
** Casepase-11 knockout ameliorate APAP-induced liver injury in mice. A** Western blot analysis of liver tissue lysates from WT mice treated with saline or APAP with antibodies against Caspase-11, GSDMD and β-actin (Results from three independent experiments, n=3). **B** Histopathological scores of the liver tissues from WT and *Casp11*^-/-^ mice (n=5), the pathologic changes of liver tissues were evaluated according to histopathologic grading. **C** Quantification of necrotic areas in liver tissues from WT and *Casp11*^-/-^ mice (n=5). **D** Hematoxylin and eosin (H&E) staining of the liver tissue biopsies from WT and *Casp11*^-/-^ mice, showing necrosis in the liver (images are representative of five independent experiments, Scale bars represent 50 μm). **E~G** Levels of serum AST, ALT, and T.BIL in WT and *Casp11*^-/-^ mice (n=5) at 0, 6, 12, 18, 24h after APAP treatment, measured by ELISA assay. **H** Western blot analysis of liver tissue lysates from WT and *Casp11*^-/-^ mice treated with APAP for 0, 1, 2, 4h, probed with antibodies against CYP2E1, β-actin, p-JNK, JNK (n=3). **I** Relative protein levels of CYP2E1 (n=3). **J** Relative mRNA expression levels of CYP2E1 (n=4). **K** Relative protein level of p-JNK/JNK (n=3). (*p < 0.05, **p<0.01, ***p < 0.001, ****p<0.0001, compared to the other group at the same time, n=3 means representative of at least three independent experiments).

To explore the effects of Caspase-11 in AILI, we detected its protein level in the liver of mice treated by APAP. As shown in Figure 1A, liver Caspase-11 was significantly upregulated and activated after APAP treatment, and cleavage of GSDMD also demonstrated activation of Caspase-11. This result suggested a positive correlation between Caspase-11 and AILI.

To further evaluate the potential effect of Caspase-11 in AILI, we used Caspase-11 knock-out mice (*Casp11*^-/-^ mice) and, as previously described, administered 350 mg/kg APAP intraperitoneally to induce AILI^26^ (Fig. S1). The HE staining of liver biopsy indicated that compared to wild-type mice (WT mice), APAP-induced liver damage was significantly alleviated in *Casp11*^-/-^ mice at 6, 12, 18, and 24h after APAP treatment (Fig. 1D). This was also reflected in the reduced necrosis area and lower pathological score in *Casp11*^-/-^mice (Fig. 1B, C). Furthermore, we found a significantly reduced serum liver injury markers, such as aspartate aminotransferase (AST), alanine aminotransferase (ALT), and total bilirubin (T.BIL), in APAP-treated *Casp11*^-/-^ mice. These results indicated that knockout of Caspase-11 may mitigate the APAP-induced liver damage (Fig. 1E, F, G).

AILI has different stages of progression. During the metabolism stage of AILI (0-4h), CYP450-mediated metabolism of excess APAP forms the reactive metabolism-NAPQI, leading to mitochondrial oxidative stress and activation of the JNK signaling pathway, resulting in damage of hepatocytes^37^-^41^. To determine if Caspase-11 affects the early stage of AILI, we measured CYP2E1 at the mRNA and protein levels. However, we found that there were no significant differences between *Casp11*^-/-^ mice and WT mice at 1h, 2h, and 4h after APAP administration (Fig. 1H, I, J). Additionally, p-JNK levels at 1h, 2h, and 4h after APAP treatment were comparable between Caspase-11 knockout mice and WT mice (Fig. 1H, K). The results above indicated that Caspase-11 does not exacerbate liver injury by affecting early hepatocyte-autonomous events and Caspase-11 is likely not to affect the primary liver injury of AILI.

### Deletion of Caspase-11 inhibited APAP-induced inflammation in liver

**Figure 2:**
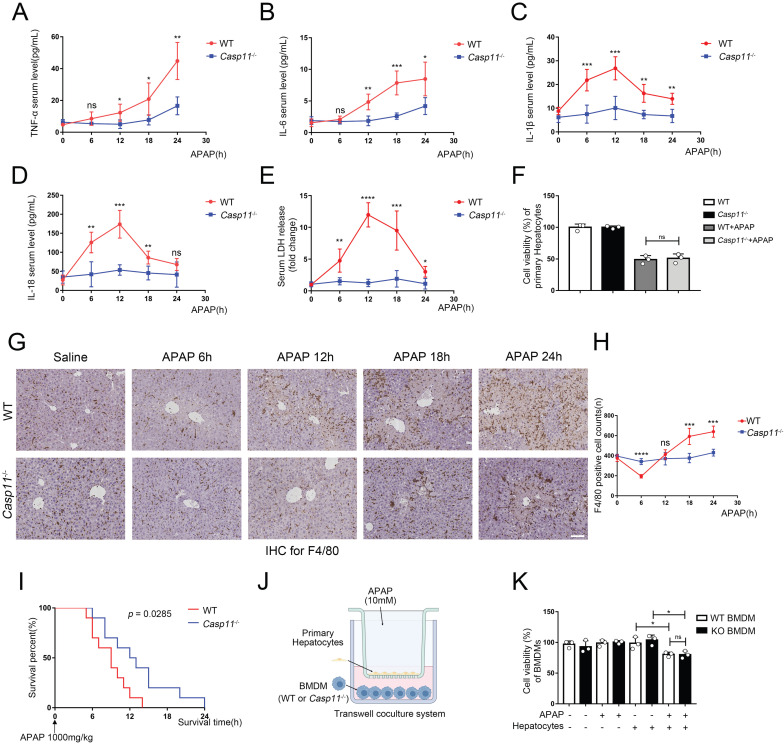
** Deletion of Caspase-11 inhibited APAP-induced inflammation and macrophage depletion in liver. A-E** Serum levels of TNF-α, IL-6, IL-1β, IL-18, and LDH in WT and *Casp11*^-/-^ mice at 0, 6, 12, 18, 24h after APAP treatment, measured by ELISA (n=5). **F** Cell viability of primary hepatocytes was measured with CCK-8 assay. **G** Images of F4/80 staining of liver tissues from WT and *Casp11*^-/-^ mice after treatment with APAP for 0, 6, 12, 18, 24h (images are representative of five independent experiments, scale bars represent 50 μm, each group contains 5 mice). **H** F4/80 positive cell counts in each field of liver tissue from WT and *Casp11*^-/-^ mice, showing survival of macrophages (n=5). **I** Kaplan-Meier survival curves comparing the survival rate of WT and *Casp11*^‑/-^ mice treated with 1000 mg/kg APAP (n = 10 for each group). **J** Experimental scheme of APAP treatment strategy in primary hepatocytes and WT or *Casp11*^-/-^ BMDMs. Primary hepatocytes were seeded into the upper chambers and treated with APAP (10 mM) for 24 h. Then hepatocytes were co-cultured with BMDMs seeded in the bottom chambers for 24 h. **K** Cell viability of WT or *Casp11*^-/-^ BMDMs was measured with CCK-8 assay (n=3). (*p < 0.05, **p<0.01, ***p < 0.001, ****p<0.0001, compared to the other group at the same time, n=3 means representative of at least three independent experiments).

Inflammation is thought to mediate the second phase of AILI^42^. Since *Casp11*^-/-^ didn't affect the metabolism and early injury stage of AILI, we next investigated whether deletion of Caspase-11 affects the severity of AILI by alleviating the inflammatory response at the 6~24h of AILI. The detection of the most common pro-inflammatory factors TNF-α and IL-6 in mice serum showed no statistical differences between *Casp11*^-/-^ mice and WT mice at 6h. However, at 12h, 18h, and 24h, serum TNF-α and IL-6 in *Casp11*^-/-^ mice were significantly lower than those in WT mice (Fig. 2A, B). Assays on liver tissue showed consistent results, with significantly lowered levels of TNF-α and IL-6 in *Casp11*^-/-^ mice than in WT mice at each time point (Fig. S2 A, B). Of note, serum TNF-α and IL-6 were significantly higher than the baseline levels at each time point, which may be related to the inevitable inflammatory response and hepatocyte damage after liver injury^43^. Besides, we measured the serum levels of two inflammasome-mediated inflammatory cytokines, IL-1β and IL-18, which were reported to be associated with AILI^44^. Serum IL-1β and IL-18 were significantly increased at 0-12h after APAP administration and decreased at the subsequent 12-24h in WT mice, while IL-1β and IL-18 were just mildly elevated in *Casp11*^-/-^ mice (Fig. 2C, D). Consistently, the levels of IL-1β and IL-18 in liver homogenates showed similar results as in serum (Fig S2 C, D). The results above suggested that the inflammatory response mediated by inflammasome may be suppressed in *Casp11*^-/-^ mice. TNF-α and IL-6 are indicative of inflammatory responses. In *Casp11*^-/-^ mice, reduced macrophage pyroptosis, particularly the diminished release of pro-inflammatory factors such as IL-1β, mitigates secondary liver damage, leading to a reduction in the overall inflammatory response. Consequently, levels of TNF-α and IL-6 are also decreased. Since Caspase-11 is highly relevant to non-canonical inflammasome activation and downstream pyroptosis, and cell rupture during pyroptosis releases abundant lactate dehydrogenase (LDH) 45, 46, we then measured LDH both in serum and in liver homogenates. In WT mice, LDH levels increased rapidly both in serum and liver homogenates at 0-12 h, then decreased briskly to baseline levels at 12-24 h. By contrast, LDH in *Casp11*^-/-^ mice did not show significant changes from baseline (Fig. 2E, Fig. S2E). Collectively, these results indicated that Casp11-dependent pyroptosis was hindered in *Casp11*^-/-^ mice. We then questioned whether suppressed Casp11-dependent pyroptosis in *Casp11*^-/-^ mice was mediated by hepatocytes. *In vitro* experiments exhibited no significant differences in the viability of WT and *Casp11*^-/-^ hepatocytes after 24h of APAP treatment (Fig. 2F), which further confirmed that knockout of Caspase-11 did not mitigate APAP-induced liver injury by directly protecting hepatocytes.

### Deletion of Caspase-11 inhibited APAP-induced macrophage depletion in liver

Macrophages are one of the main sources of IL-1β and IL-18, and the role of macrophages in AILI have not been fully elucidated^30^, ^47^. Hence, we performed immunohistochemistry (IHC) with F4/80 monoclonal antibody to determine the viability of macrophages in the liver (Fig. 2G, H). There were significantly reduced F4/80^+^ cells in the livers of WT mice at 6 hours after APAP treatment, and returned to baseline level at 12h, then elevated substantially from baseline level at 18-24h. Conversely, in Casp11 KO mice, the number of macrophages did not change significantly after APAP treatment, which suggested that knockout of Caspase-11 may exert effectiveness through a macrophage-mediated pathway. This suggests that macrophages may undergo cell death through a Casp11-dependent pathway, and the knockout of Casp11 weakens macrophage recruitment induced by inflammation. the reduction in the number of F4/80^+^ cells at the 6-hour time point may be attributed to pyroptosis of macrophages. Subsequent inflammatory reactions recruit circulating macrophages, leading to an increase in their numbers. The elevated levels of IL-1β and IL-18 are closely associated with the activation of inflammasomes and cellular pyroptosis. Next, we treated WT and *Casp11*^-/-^ mice with lethal doses(1000mg/kg) of APAP to determine the prognosis, and Kaplan-Meier curves showed *Casp11*^-/-^ mice had better survival with statistical significance (*p*=0.0285, Fig. 2 I). Meanwhile, nearly all WT mice died within 6-12 hours while 50% of *Casp11*^-/-^ mice survived at 12 hours. To sum up, these results suggested that there is a drastic Caspase-11-dependent inflammatory response associated with macrophage death, which could be attenuated by knockout of Caspase-11, and attenuation of this inflammatory response could reduce macrophage death and alleviate liver damage caused by APAP.

To further determine whether APAP-treated hepatocytes have an inhibitory effect on macrophages, we established an *in vitro* co-culture system (Fig. 2J). After co-cultured with hepatocytes, the viability of bone marrow-derived macrophages (BMDMs) was lower than the control group. It might result from various substances (such as DAMPs) released from damaged hepatocytes. However, the differences in viability were not statistically significant between treated WT and *Casp11*^-/-^ BMDMs (Fig 2K). The further detection of macrophage LDH release obtained comparable results as cell viability that APAP-induced relative LDH release in supernatants of BMDMs only occur when co-cultured with hepatocytes regardless the Caspase-11 knockout status in macrophages (Fig. S2F). This result indicates that it may be necessary to further explore the function of Caspase-11 in macrophages.

### Non-canonical pathway mediated by Caspase-11 promoted APAP-induced liver injury

Given that LPS is considered to be the activator of Caspase-11, and the roles of LPS and LPS-induced non-canonical pyroptosis in AILI have not been elucidated, we next explored the role of LPS-induced non-canonical pyroptosis in AILI^2^, ^4^, ^48^. First, we found that the levels of LPS in the portal circulation of either WT mice or *Casp11*^-/-^mice were significantly increased at 6h after APAP challenge, and then returned to baseline at the subsequent 12-24h. (Fig. S3A). Moreover, immunofluorescence confocal experiments with LPS and F4/80 staining showed identical results that the number of LPS spotted in liver tissue was significantly increased at 6h after APAP administration, and decreased afterwards (both in WT or *Casp11*^-/-^ mice). Consistent with our previous assumption, the F4/80 staining representing m acrophages decreased significantly at 6h and gradually increased later (LPS here may originate from a dysregulated gut microbiota^26^). We also observed the LPS within the cytoplasm of F4/80^+^ cells, which provides a necessary condition for activation of non-canonical pyroptosis in macrophages (Fig. S3B).

**Figure 3:**
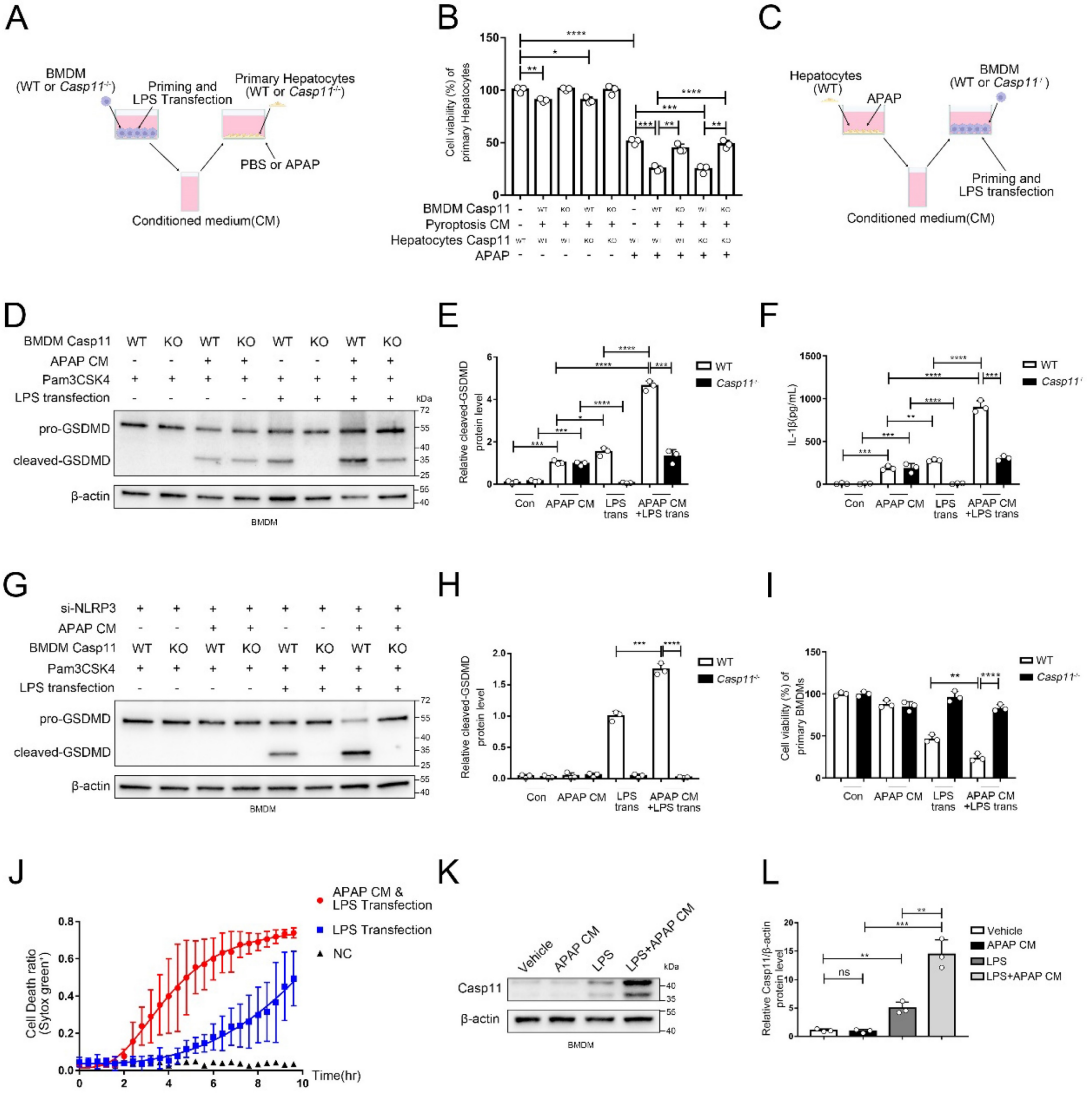
** Non-canonical pathway mediated by Caspase11 promote APAP-induced liver injury**. **A** Experimental scheme of treating strategy in BMDMs (WT or *Casp11*^-/-^) and primary hepatocytes (WT or *Casp11*^-/-^). Hepatocytes were cultured with CM of BMDMs with an additional APAP (10 mM) for 24h. **B** Cell viability of WT or *Casp11*^-/-^ hepatocytes was measured with CCK-8 assay (n=3). **C** Experimental scheme of treating strategy in BMDMs (WT or *Casp11*^-/-^) and primary hepatocytes (WT). BMDMs were cultured with CM of hepatocytes treated by APAP (10 mM) for 24h, then priming by Pam3CSK4(1ug/mL) for 4h and transfection ultra-pure LPS(1ug/mL) for 6h to trigger non-canonical pyroptosis.** D-E** Cell lysate of BMDMs was analyzed by Western blot using antibodies against GSDMD and β-actin(n=3). The relative cleaved-GSDMD level compared with β-actin was shown as a line graph. **F** IL-1β in cell supernatants were analyzed by ELISA assay. **G-H** Cell lysate of BMDMs was analyzed by Western blot using antibodies against GSDMD and β-actin(n=3). Relative cleaved-GSDMD level compared with β-actin was shown as a line graph. **I** Cell viability of BMDMs was measured with CCK-8 assay (n=3). **J** BMDM Cell death kinetics were monitored over time following inflammasome activation by measuring the incorporation of SYTOX Green. **K-L** Cell lysate of BMDMs treated by PBS, APAP CM, LPS, and LPS+APAP CM were analyzed by Western blot using antibodies against Caspase-11 and β-actin(n=3). Relative Casp11 level compared with β-actin was shown as a line graph. (*p < 0.05, **p<0.01, ***p < 0.001, ****p<0.0001, compared to the other group at the same time, n=3 means representative of at least three independent experiments).

In view of that the activation of non-canonical pyroptosis drastically affected inflammatory response, which may lead to a secondary injury in AILI, we further scrutinized whether non-canonical pyroptosis engaged in the process of AILI. We applied Pam3CSK4(TLR1/2 Agonist) as priming signal followed by LPS transfection to induce non-canonical pyroptosis of macrophages, and collected its conditioned medium (CM). Then we added CM to the hepatocyte culture system with or without APAP to study its effects on hepatocytes after 24 hours exposure (Fig. 3A). The viability of hepatocytes was decreased when CM was used alone, which could be reversed by Caspase-11 knockout on BMDMs but not on hepatocytes. When adding both CM and APAP, we observed substantially aggravated APAP-induced hepatocyte death which, as previously, could be reversed by Caspase-11 knockout on BMDMs but not on hepatocytes (Fig. 3B). Collectively, these results suggested that APAP-induced liver damage could be exacerbated by Caspase-11-mediated non-canonical pyroptosis.

### Microenvironment of AILI up-regulates non-canonical pyroptosis

So far, we have confirmed a critical role for non-canonical pyroptosis in APAP-induced liver injury, while the role of AILI on non-canonical pyroptosis has not been elucidated. In this way, we applied hepatocytes with APAP for 24 hours and collected the supernatant as conditioned medium (CM) to treat Pam3CSK4-primed macrophages. Then, we transfected ultra-pure LPS into macrophages to specifically induce non-canonical pyroptosis (Fig. 3C). Cleaved-GSDMD, which is generated by cleavage of pro-GSDMD by Caspase-11, is an effector molecule of pyroptosis and one of the gold standards for detecting the level of pyroptosis^46^, ^49^. Therefore, we assessed the level of macrophage pyroptosis by detecting the level of cleaved-GSDMD by Western blot (Fig. 3D, E). A low-leveled cleavage of GSDMD was observed in BMDMs treated with APAP CM only without LPS transfection, and knockout of Caspase-11 can't reverse it (lane 3,4 vs. lane 1,2). Similar results were obtained for the detection of IL-1β released by cells in Fig. 3F. (Casp11 can also activate NLRP3 through non-canonical pathways to mature IL-1β). The previous result reflects that APAP CM directly activates canonical pyroptosis in BMDMs through the oxidative stress pathway^50^. GSDMD in macrophages was significantly activated by LPS transfection, which could be completely inhibited by Caspase-11 knockout (lane 5,6). Intriguingly, the cleavage of GSDMD was markedly enhanced when treating BMDMs with APAP CM during the priming phase, and this effect could also be completely reversed by Caspase-11 knockout (lane 5,7,8). To exclude the impact of the NLRP3/ASC/Caspase-1 pathway, we repeated the experiment of Fig. 3C with BMDMs cell line silencing of NLRP3 by siRNA (Fig.S3C). We observed that APAP CM still promote the cleavage of GSDMD in non-canonical inflammasome activity (Fig. 3G, H), confirming the specific activation of non-canonical pyroptosis pathway by APAP CM. Fig 3 D and G data showed NLRP3 is required for APAP CM induced GSDMD cleavage, and knockdown of NLRP3 will lead to weakening of GSDMD cleaved. Same results were obtained by cell viability assay (Fig. 3I). The results of Fig. 3J also proved that BMDMs exhibited a faster death kinetics under the condition of APAP CM treatment. The above evidence en masse suggested that the microenvironment of APAP CM could intensify the non-canonical pyroptosis of macrophages.

To clarify how APAP CM exerts its effect, we measured the protein level of Caspase-11 in macrophages. We found that APAP CM alone is not sufficient to upregulate Caspase-11, but strikingly, when act together with LPS, it significantly increased Caspase-11 protein level in macrophages (Fig. 3K, L). Together with previous results, this implied that the microenvironment of AILI may intensify the non-canonical pyroptosis of macrophages by regulating Caspase-11.

### Lactate in microenvironment of AILI upregulates non-canonical pyroptosis

**Figure 4:**
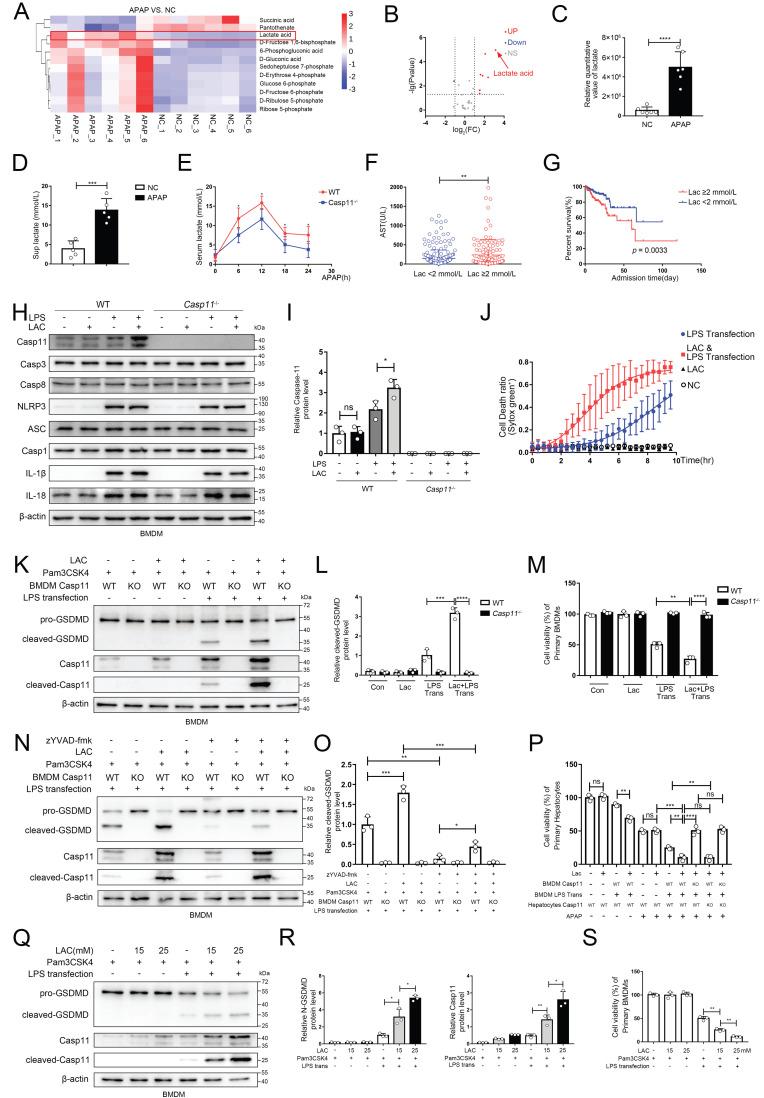
** Lactate in microenvironment of AILI upregulates non-canonical pyroptosis. A** Heatmap of differential metabolites analyzed by metabolomics of BMDMs co-culture with hepatocytes treated by PBS, APAP (10 mM) for 24h. The transverse axis represents the clustering of samples and longitudinal axis represents the clustering of metabolites (n=6). **B** Volcano plot showing differential metabolites. **C** Relative quantitative value of lactate. **D** Lactate levels in cell supernatants were analyzed by ELISA assay(n=5). **E** Serum lactate levels among WT and *Casp11*^-/-^ mice after being treated by APAP 0, 6, 12, 18, and 24h were measured by ELISA assay(n=5). **F** Serum AST levels among DILI patients were divided into 2 groups according to their lactate levels. These patients were diagnosed from August 2011 to June 2022 in the first affiliated hospitals of Xi'an Jiaotong University. **G** Kaplan-Meier curve for hospital survival stratified by lactate level. **H-I** Cell lysate of WT and *Casp11*^-/-^ BMDMs treated by PBS, LAC (25mM), LPS (500 ng/mL), and LPS+LAC were analyzed by Western blot using antibodies against Caspase-11, Casp3, Casp8, NLRP3, ASC, Casp1, IL-1β, IL-18 and β-actin(n=3).** J** BMDM Cell death kinetics were monitored over time following inflammasome activation by measuring the incorporation of SYTOX Green. **K-L** Cell lysate and supernatants of BMDMs was analyzed by Western blot using antibodies against GSDMD, Casp-11 and β-actin (n=3). Relative cleaved-GSDMD level compared with β-actin was shown as a line graph. **M** Cell viability of WT or *Casp11*^-/-^ BMDMs was measured with CCK-8 assay (n=3). **N-O** Cell lysate and supernatants of BMDMs was analyzed by Western blot using antibodies against GSDMD, Casp-11 and β-actin (n=3). Relative cleaved-GSDMD level compared with β-actin was shown as a line graph. **P** Cell viability of WT or *Casp11*^-/-^ hepatocytes was measured with CCK-8 assay (n=3).** Q-R** Cell lysate and supernatants of BMDMs was analyzed by Western blot using antibodies against GSDMD, Casp-11 and β-actin (n=3). Relative cleaved-GSDMD level compared with β-actin was shown as a line graph. **S** Cell viability of WT or *Casp11*^-/-^ hepatocytes was measured with CCK-8 assay (n=3). (*p < 0.05, **p<0.01, ***p < 0.001, ****p<0.0001, compared to the other group, n=3 means representative of at least three independent experiments, etc.).

Recently, it has been reported that metabolites could affect the process of pyroptosis, and NAPQI, a reactive metabolite of APAP, could vigorously induce mitochondrial dysfunction and cause metabolic disorders in hepatocytes, but whether macrophage metabolism is affected during AILI remains unclear^8^, ^41^, ^51^. We used the same experimental protocol as Fig. 2J, and identified central carbon metabolism of macrophages by metabolomics^52^, ^53^. Then, we screened the differential metabolites between the NC group and the APAP group (Fig. 4A demonstrated the results of clustering analysis of the differential metabolites between two groups). Results revealed that the levels of substances represented by lactic acid were significantly increased in the APAP group, whilst the levels of substances represented by succinic acid were significantly decreased. The volcano plot (Fig. 4B, C) demonstrated that lactate was the most variable metabolites among the differential metabolites screened. Additionally, we found that lactate levels in supernatants of cell culture were also significantly increased after APAP interference through ELISA (Fig. 4D). We also determine the lactate levels in the supernatant from hepatocytes treated with APAP without macrophage, this result revealed a significant increase in lactate levels in the supernatant. However, they appeared to be lower than the lactate levels in the co-culture system, possibly due to the interaction between the two types of cells (Fig.S4A). To ascertain whether APAP CM and LPS transfection treatment could upregulate lactate levels in the cytoplasm of macrophages, we measured the relative changes in lactate levels in the cytoplasm under both treatments. After both treatments, the lactate levels in macrophages increased, but the lactate levels in the APAP CM treatment group were relatively higher (Fig. S4B, C). At animal level, we found mice serum lactate increased rapidly in the initial 12h after APAP treatment, and then gradually decreased, while less serum lactate was observed in *Casp11*^-/-^ mice at each time point (Fig 4E). In brief, lactate was significantly upregulated intracellularly, extracellularly in AILI. Since lactate is widely used as a predictive marker in various diseases 54, clinical information of AILI patients was collected and analyzed. Patients who had higher lactate levels tended to have more severe liver damage, as well as much worse prognosis (Fig. 4F, G; Fig. S4D, E). Intriguingly, serum lactate level of AILI patients is also positively correlated with MELD score, an important prognostic indicator for liver transplantation patients (Fig S4 F). Next, we confirmed that LPS is essential for lactate to upregulate the level of Casepase-11 in macrophages, However, other pyroptosis-related proteins (NLRP3, ASC, Casp1, IL-1β, IL18, Casp3, Casp8, AIM2, pyrin, NLRC4) were not regulated by lactate (Fig. 4 H, I and Fig. S4 G). Then, we stimulated BMDMs with lactate when giving priming signal, followed by LPS transfection to induce non-canonical pyroptosis. Cell death kinetics demonstrated a significant increase in the death rate with lactate interference (Fig. 4 J). Moreover, an elevation of the cleaved-GSDMD was observed after lactate stimulation, which could be reversed completely by caspase-11 knockout, indicating that lactate upregulates non-canonical pyroptosis of macrophages in a casepase-11-dependent manner (Fig. 4 K, L). The results of cell viability assay showed similar findings (Fig. 4 M). To mitigate the potential influence of NLRP3 activation on the results, we employed a specific inhibitor of Casp1, zYVAD-fmk, to suppress its activity. The outcomes showed that although zYVAD-fmk weakened the GSDMD cleavage, lactate continue to enhance the cleavage level of GSDMD after LPS transfection, indicating that the strengthening effect of lactate on pyroptosis was specific to Casp11 (Fig. 4 N, O). To determine the dose-response relationship with lactate, we subjected BMDMs to varing concentrations of lactate to induce non-canonical pyroptosis. We observed a progressive increase in non-canonical pyroptosis and a concomitant rise in the protein level of Casp-11 as the lactate concentration escalated (Fig. 4 Q, R). Subsequent cell viability measurements also confirmed this phenomenon (Fig. 4S). Eventually, we re-examined the effect of lactate on AILI through the same protocols described in Fig 3A. In Fig. 4P, by comparing Ctrl-CM with LAC-only CM, we found that the addition of LAC alone did not affect the viability of hepatocytes, nor did it affect the degree of APAP inhibition on hepatocyte viability (lane 1,2 and lane 5,6). However, it was found that LAC+LPS trans CM could result in a more destructive effect of APAP on hepatocytes compared to LPS trans CM (lane 8 vs. lane 7). Additionally, the effect of LAC could be completely reversed by caspase-11 knockout in BMDMs (lane 9,11). Taken together, these results suggested that lactate aggravated the non-canonical pyroptosis of macrophages by up-regulating caspase-11, leading to an exacerbation of APAP-induced liver injury.

### Lactate reduce the ubiquitination of the Caspase-11 by decreasing the binding affinity of NEDD4 and Caspase-11

**Figure 5:**
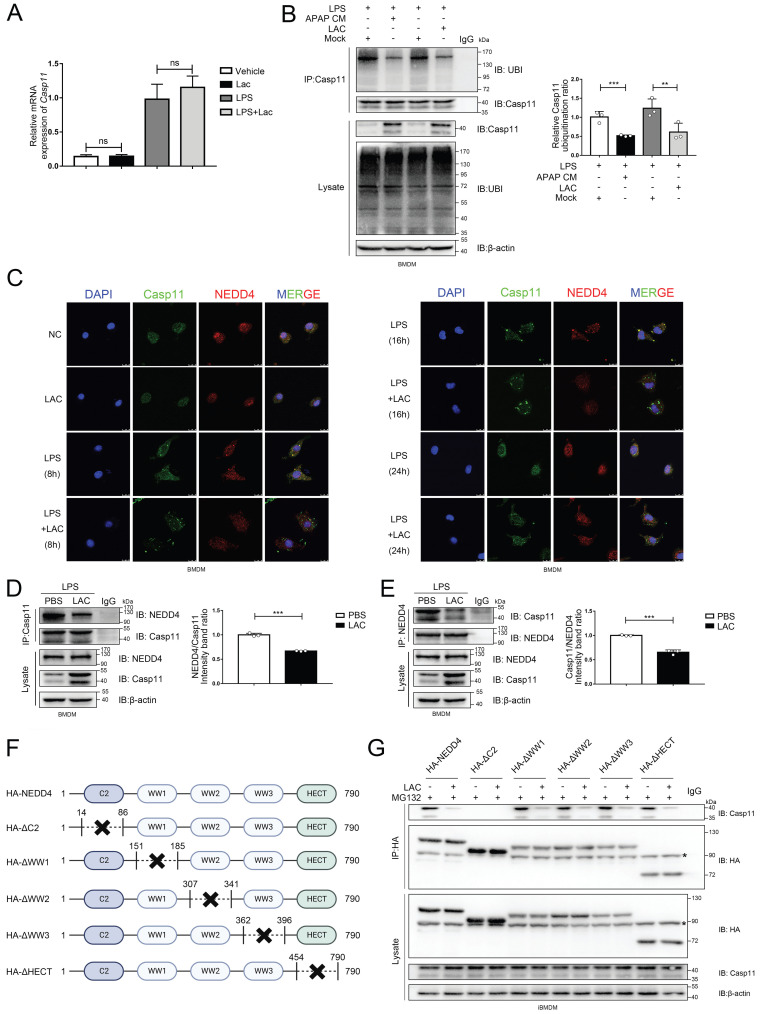
** Lactate reduce the ubiquitination of the Caspase-11 by decrease the combination of NEDD4 and Caspase-11. A** mRNA levels of Casp11 in BMDMs treated by LPS, LPS+APAP CM, and LPS+LAC measured by qPCR assay(n=3). **B** BMDMs were incubated with LPS (500 ng/mL) for 4h, lysed, and immunoprecipitated with anti-Caspase-11 antibody. Both the immunoprecipitates (IP) and whole cell lysates (WCL) were subjected to gel electrophoresis and immunostaining with an anti-Ubi or anti-Caspase-11 antibody(n=3). **C** Images of immunofluorescence staining of the BMDMs treated by PBS, LPS, LPS, and LAC. NEDD4 (red) and Caspase-11 (green) were examined by confocal microscope. The nucleus was indicated by DAPI (blue) staining. **D-E** BMDM cells were incubated with LPS for 4 h, lysed and immunoprecipitated with anti-Caspase-11 or anti-NEDD4 antibody. Both the immunoprecipitates (IP), and whole cell lysates (WCL) were subjected to gel electrophoresis and immunostaining with an anti-NEDD4 or anti-Caspase-11 antibody(n=3). **F** Schematic representation of full-length NEDD4 or indicated mutants. **G** Coimmunoprecipitation assay to assess the interactions between Casp11 and NEDD4 or its truncation mutants. (*p < 0.05, **p<0.01, ***p < 0.001, ****p<0.0001, compared to the other group, n=3 means representative of at least three independent experiments).

Non-canonical pyroptosis is directly regulated by Caspase-11^6^. To verify whether lactate modulates the transcription of Caspase-11, we detected the mRNA level of Caspase-11 in macrophages after lactate treatment by qPCR (Fig. 5A). Surprisingly, lactate didn't affect the Caspase-11 transcription regardless of the presence of LPS. Ubiquitination is one of the most common post-translational modifications, which directs protein degradation through the proteasome, and plays an important role in inflammation and immune cells^55^, ^56^. Therefore, we identified the impact of APAP CM and LAC on the caspase-11 ubiquitination under the interference of LPS by immunoprecipitation (IP) (Fig. 5B). The results suggested that both APAP CM and LAC can attenuate the ubiquitination of Caspase-11. This indicated that lactate may regulate the protein level of Caspase-11 by affecting ubiquitination. Previous studies reported that NEDD4 can bind to Caspase-11 and degrade it through a 26S proteasome-mediated pathway^19^, ^20^. Accordingly, we performed confocal immunofluorescence colocalization experiments to explore whether lactate affects the binding of NEDD4 to Caspase-11. As shown in figure 5C, LPS (500 ng/mL) alone could increase the Caspase-11 in macrophages, and the green punctuates formed by Caspase-11 and the co-localized spots formed by NEDD4 and Caspase-11 could be observed at 8 and 16 h. However, the co-localization of Caspase-11 and NEDD4 was significantly dampened with the addition of lactate, and the number of Caspase-11 fluorescent spots was significantly increased. These results implied that lactate may affect the ubiquitination of Casp11 by altering the binding of NEDD4 to Casp11. Co-IP revealed that NEDD4 was able to interact with Caspase-11 in LPS-transfected macrophages, which could be attenuated by lactate administration (Fig. 5D, E). Continuing forward, as the domain responsible for mediating the interaction between NEDD4 and CASP11 is not yet clear, we truncated and mutated the domains of NEDD4 sequentially, discovering that the binding of NEDD4 to Casp11 hinges on the critical involvement of the C2 domain (Fig. 5 F, G). Collectively, these results indicated that lactate in microenvironment of AILI could reduce the ubiquitination of Caspase-11 by inhibiting the binding of NEDD4 and Caspase-11.

### Lactate directly induces NEDD4 lactylation (Klac) in macrophages

**Figure 6:**
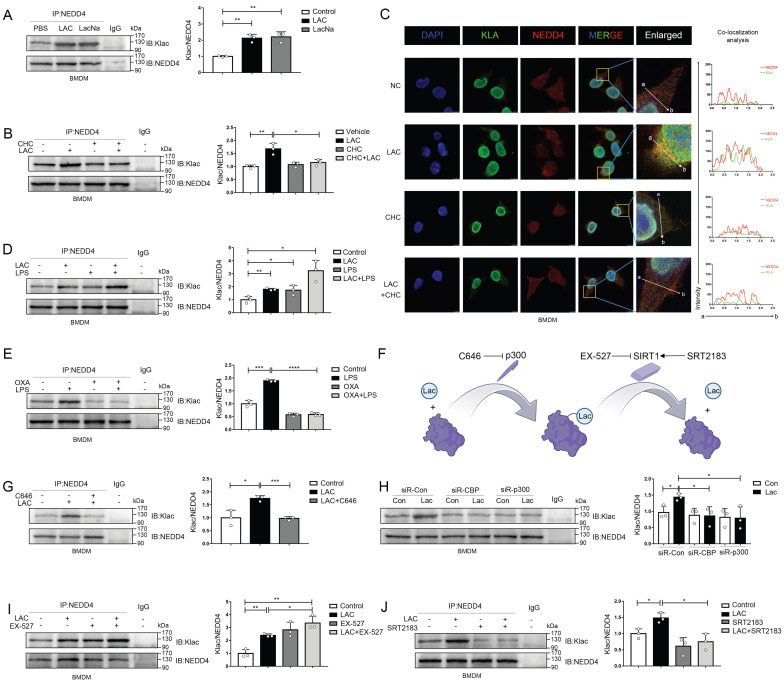
** Lactate directly induces NEDD4 lactylation (Klac) in macrophages. A** Protein lysate of lactate or sodium lactate-treated BMDMs were precipitated with anti-NEDD4 antibody followed by immunoblotting with anti-Klac antibody showing lactate-induced Klac in NEDD4 immunocomplex (n = 3). **B** BMDMs were pretreated with CHC (3 mM) or vehicle for 2 h followed by lactate (25 mM) addition for 6h. NEDD4 Klac levels were assayed by immunoprecipitation with anti-NEDD4 antibody followed by immunoblotting with anti-Klac antibody(n=3). **C** BMDMs were treated with CHC (3 mM) or DMSO for 2 h before lactate (25 mM) addition for another 6 h. Klac (green) and NEDD4 (red) co-localization was examined by confocal microscope. The nucleus was indicated by DAPI (blue) staining. Co-localization analysis was performed using Zeiss Zen microscope software. **D** BMDMs were treated with LPS (500 ng/mL) or lactate (25 mM) or both for 6h. Protein lysates were precipitated with anti-NEDD4 antibody followed by immunoblotting with anti-Klac antibody (n = 3). **E** BMDMs were pretreated with oxamate (20 mM) for 30 min followed by LPS (500 ng/mL) stimulation for 6h. Protein lysates were precipitated with anti-NEDD4 antibody followed by immunoblotting with anti-Klac antibody (n = 3). **F** Schematic diagram of NEDD4 lactylation regulated by p300 and SIRT1. **G** BMDMs were treated with C646 (5 µM) or vehicle for 2 h followed by lactate treatment for 6 h. Protein lysates were precipitated with anti-NEDD4 antibody followed by immunoblotting with anti-Klac antibody (n = 3). **H** CBP and p300 were silenced by transfection with specific siRNAs (40 nM) overnight followed by lactate (25 mM) stimulation for 6 h. Protein lysates were precipitated with anti-NEDD4 antibody followed by immunoblotting with anti-Klac antibody (n = 3) **I** Suppression of SIRT1 deacetylase activity by EX-527 (10 μM) increased NEDD4 lactylation in BMDMs (n = 3). **J** Activation of SIRT1 deacetylase by its activator SRT2183 (10 μM) decreased lactylated-NEDD4 in BMDMs (*n* = 3). (*p < 0.05, **p<0.01, ***p < 0.001, ****p<0.0001, compared to the other group, n=3 means representative of at least three independent experiments).

NEDD4 is a HECT E3 ubiquitin ligase, and previous studies illustrated that the activity of this type of ubiquitin ligase could be regulated by post-translational modifications such as acetylation^22^, ^57^. Moreover, several recent studies have shown that lactate can directly modify a variety of proteins, leading to a protein modification-lactylation, to regulate protein functions^13^, ^15^. By IP assay, we found that exogenous lactate administration and sodium lactate could equally promote the lactylation of NEDD4 in macrophages (Fig. 6A). Next, we used CHC, an inhibitor of MCT1 (monocarboxylate transporter), to inhibit the entry of exogenous lactate into cells and found that the use of CHC effectively blocked lactylation of NEDD4 induced by exogenous lactate (Fig. 6B)^58^. Immunofluorescence staining also revealed that lactate promoted the co-localization of Klac and NEDD4 in BMDMs, which could be abrogated by CHC (Fig. 6C). Since LPS has been reported to upregulate histone lactylation in macrophages, we scrutinized whether LPS could regulate lactylation of NEDD4 (Fig. 6D). As expected, we found that LPS was able to upregulate lactylation of NEDD4 and have a synergistic effect with exogenous lactate administration. This suggested that LPS may regulate lactylations through an endogenous pathway. Thus, we inhibited endogenous lactate production by oxamate (OXA)^59^ and stimulated macrophages simultaneously with LPS. We found that OXA was able to inhibit LPS-induced lactylation of NEDD4 (Fig. 6E). Lysine acetylase p300 has been reported to catalyze the transfer of the lactyl-group from lactyl-CoA to histones in a cell-free system^13^. Consequently, we attempted to determine whether p300 or its homolog, CBP, can drive the lactylation of NEDD4 in macrophages. To this end, we inhibited the activity of p300 by C646^60^ and observed a significantly reduced lactylation of NEDD4 (Fig. 6G).

Correspondingly, silencing of p300 or CBP by siRNA also attenuated lactate-induced NEDD4 lactylation in macrophages (Fig. 6H). SIRT1, a candidate protein delactylase, has been reported to bind directly to NEDD4^61^-^63^. Hence, we used EX-527, a specific SIRT1 inhibitor, to interfere the SIRT1 in macrophages. As expected, the inhibition of SIRT1 could upregulate NEDD4 lactylation and possessed a synergistic effect with exogenous lactate (Fig. 6I). Likewise, the activation of SIRT1, induced by a specific SIRT1 agonist-SRT2183, significantly suppressed the NEDD4 lactylation in macrophages (Fig. 6J). Finally, we validated in a cell-free system that P300 can facilitate the transfer of Lac-CoA to NEDD4, leading to its lactylation modification (Fig. S5 A). Taken together, these results confirmed that both endogenous and exogenous lactate could modulate the lactylation of NEDD4 in macrophages with p300/SIRT1 to be the lactylating and delactylating enzyme, respectively (Fig. 6F).

### Lactylation of NEDD4 impairs its ability to ubiquitinate Caspase-11

**Figure 7:**
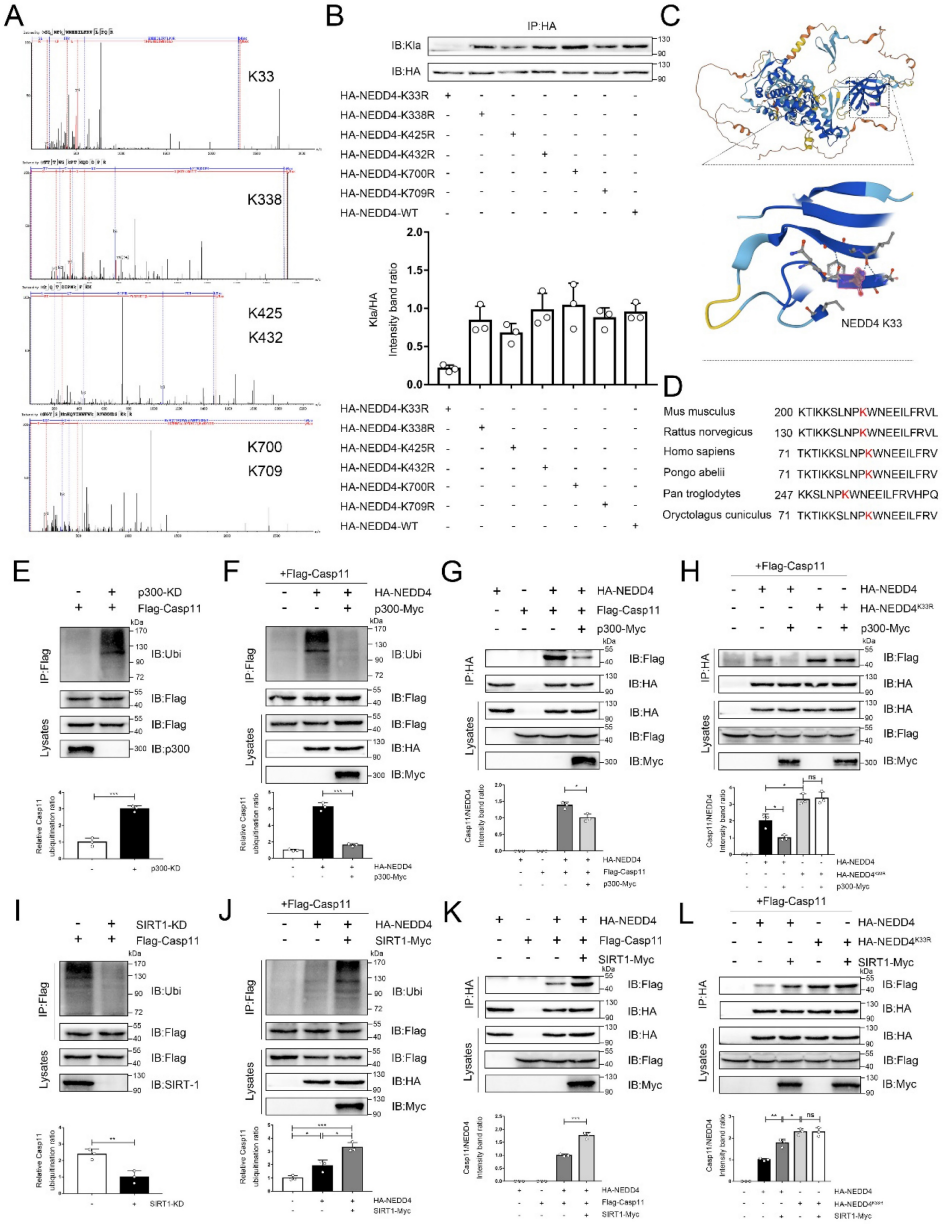
** Lactylation of NEDD4 impairs its ability to ubiquitinate Caspase-11. A** Identification of NEDD4 lactylation sites by LC-MS/MS. **B** K33R, K338R, K425R, K432R, K700R, K709R site mutations, or wild-type HA-NEDD4 overexpressed 293T cells were constructed respectively through overexpression plasmid. Cell proteins were analyzed by western blotting (WB) for HA-NEDD4 levels. **C** Ribbon diagram of the crystal structure of Mouse NEDD4 protein (AlphaFold entry AF-P46935-F1). **D** The K33 site in NEDD4 is conserved. The sequences around NEDD4 K33 from different species were aligned. Conserved lysine residues corresponding to mouse NEDD4 K33 are marked in red. **E-F** Endogenous ubiquitination of Caspase-11 were measured in cells transfected with the indicated plasmid combinations by overexpressing P300-Myc and knock-down endogenous P300 in iBMDM cells.** G-H** The influence of P300 on Caspase-11-NEDD4 interaction or Caspase-11-NEDD4K33R was determined by overexpressing the indicated plasmid combinations. **I-J** Endogenous ubiquitination of Caspase-11 was measured in cells transfected with the indicated plasmid combinations by overexpressing SIRT1-Myc and knock-down endogenous SIRT1 in iBMDM cells.** K-L** Influence of SIRT1 on Casp11-NEDD4 interaction or Casp11- NEDD4K33R interaction was determined by overexpressing the indicated plasmid combinations in cells. MG132 was used in the above experiments to inhibit protein degradation. (*p < 0.05, **p<0.01, ***p < 0.001, ****p<0.0001, compared to the other group, n=3 means representative of at least three independent experiments).

As previously noted, epigenetic modifications of NEDD4 would affect its function, but the impact of lactylation, which is induced by lactate, on NEDD4's ability to ubiquitinate Caspase-11 remained unclear. To address this, we employed the LC-MS technique to identify the lactylation site of NEDD4, and we see a mass shift of 72.02 at lysine on the peptides, and that correlated to a lactylated ms/ms spectra, according to previous studies (Associated information was shown in Fig. S6 B-D)^13^. We noted 6 sites (K33, K338, K425, K432, K700 and K709) with the most significant increase in MS peak intensity after the intervention of lactate (Fig. 7A). Then, by ectopically overexpressing HA-tagged NEDD4 into 293T cells through overexpression plasmid, we constructed K33R, K338R, K425R, K432R, K700R and K709R site mutations or WT NEDD4 overexpression 293T cells. Next, we used a HA antibody to pull down NEDD4 to detect lactylation levels. The results confirm that the K33R site mutation significantly reduced NEDD4 lactylation, these results suggest that the K33 site is the major lactylation site of HA-NEDD4(Fig. 7B). The tertiary structure of NEDD4 showed that K33 is located in the C2 domain of the NEDD4 protein (Uniport P46935) and is highly conserved across species (Fig. 7C, D, Fig. S6 A). Molecular structure simulations further revealed that K33 site is situated on the surface of the protein (Fig. S6E). It is important to note that no other post-translational modifications at this locus have been reported before. Using an ecotopic expression system and co-immunoprecipitation assays, we found that the knocking down P300 increased the ubiquitination of Caspase-11 (Fig. 7E). Conversely, the ubiquitination of Caspase-11 was significantly attenuated after P300 overexpression in iBMDM cells (Fig. 7F). Given that lactate can weaken the NEDD4's binding to Caspase-11, we theorized that lactylation decreases the Caspase-11 ubiquitination by impairing the binding of these two proteins. To verify our hypothesis, we overexpressed p300 in iBMDM cells, which resulted in a significantly weakened binding of NEDD4 to Caspase-11 (Fig. 7G). Additionally, we overexpressed HA-NEDD4 and HA-NEDD4^K33R^, the latter of which was utilized to prompt the delactylation of NEDD4, aiming to address the role of K33 site in NEDD4 lactylation. As expected, overexpression of P300 impaired the binding of wild-type NEDD4 to Caspase-11, whereas the binding of NEDD4^K33R^ to Caspase-11 was not affected (Fig. 7H). Collectively, these results indicated that lactylation of K33 site of NEDD4 could diminish its binding affinity to Caspase-11. Furthermore, when SIRT1 was knocked down, the ubiquitination of Caspase-11 was significantly decreased (Fig. 7I). In contrast, overexpression of SIRT1 significantly increased the ubiquitination of Caspase-11 (Fig. 7J). Additionally, we also observed an augmented binding ability of NEDD4 to Caspase-11 after SIRT1 overexpression (Fig. 7K), but for NEDD4^K33R^, SIRT1 overexpression would not affect its binding capacity (Fig. 7L). Interestingly, the binding capacity of NEDD4^K33R^ to Caspase-11 was stronger than that of NEDD4 which may be due to the complete elimination of endogenous lactylation for NEDD4^K33R^. Western blot data confirming the knockdown or overexpression of certain targets were shown in Fig. S6 F-K. We incubated recombinant Casp11 and P300 with HA-NEDD4, as well as HA-NEDD4^K33R^, both of which were immunoprecipitated from HEK293T cells. The results demonstrated that in the presence of Lac-CoA, P300 solely inhibit the ubiquitination of HA-NEDD4 on Casp11, while it had no effect on HA-NEDD4^K33R^ (Fig.S6 L). The results above suggest that NEDD4 K33la affects the Caspase-11 ubiquitination by altering the binding affinity of NEDD4 to Caspase-11.

### NEDD4 lactylation affects non-canonical pyroptosis

**Figure 8:**
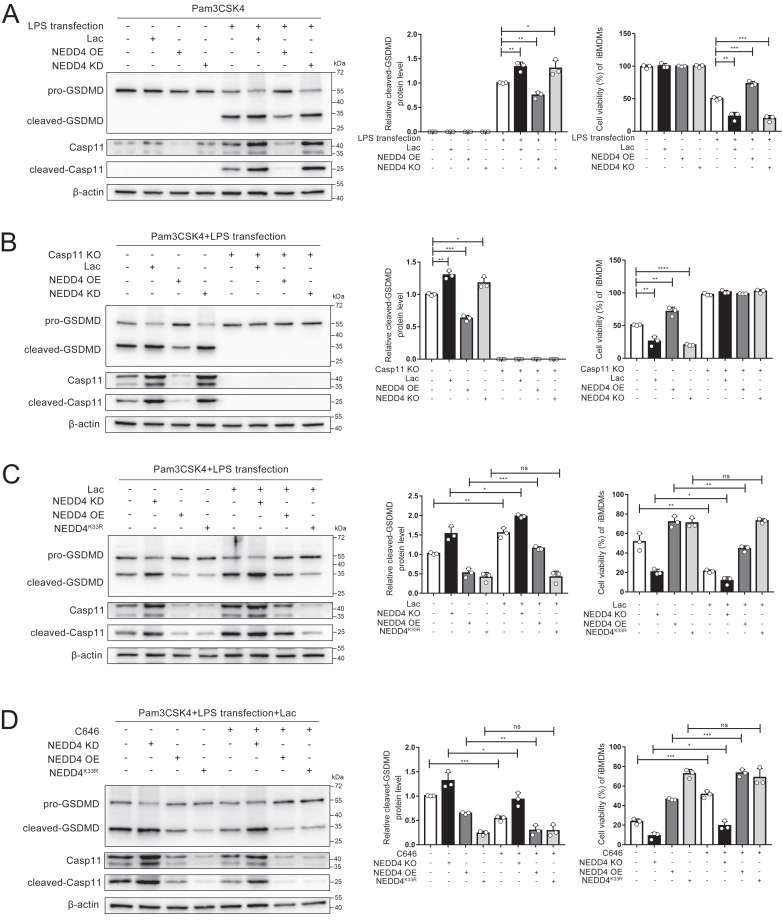
**NEDD4 lactylation affects non-canonical pyroptosis. A-D** iBMDM cells were transfected with the indicated plasmid combinations, then primed with Pam3CSK4(1 μg/mL) with or without lactate for 4h, followed by LPS transfection (1 μg/mL). Cell lysate and supernatants were analyzed by Western blot using antibodies against GSDMD, Casp11 and β-actin(n=3). The relative level of cleaved-GSDMD compared to β-actin was shown as a line graph. Cell viability of iBMDM was measured with CCK-8 assay (n=3). (*p < 0.05, **p<0.01, ***p < 0.001, ****p<0.0001, compared to the other group, n=3 means representative of at least three independent experiments).

NEDD4 has been reported to regulate non-canonical pyroptosis through the Caspase-11 pathway^20^. In this way, lactylation may regulate the NEDD4-dependent non-canonical pyroptosis. It was found that the cleavage of GSDMD was inhibited by NEDD4 overexpression and stimulated by NEDD4 knockdown, which proved that NEDD4 negatively regulates the non-canonical pyroptosis of macrophages (Fig. 8A, Fig.S7). We then knocked out caspase-11 on macrophages, which completely eliminated the regulation of lactate and NEDD4 on cleaved-GSDMD (Fig. 8B). This suggested that NEDD4 and lactate regulate pyroptosis in a Caspase-11-dependent manner. To investigate the role of NEDD4 K33la, we expressed both NEDD4 and NEDD4^K33R^ and treated them with lactate. Our results showed that lactate effectively suppressed NEDD4's negative regulation on non-canonical pyroptosis, but had no impact on non-lactylatable NEDD4^K33R^ (Fig. 8C).

Moreover, by using C646 to inhibit NEDD4 lactylation, we observed that lactate's regulation of non-canonical pyroptosis via NEDD4 was reversed (Fig. 8D). The measurement of cell viability in the corresponding iBMDMs led to the same conclusion (Fig. 8 A, B, C, D). These findings collectively demonstrate that lactate regulates non-canonical pyroptosis mediated by Caspase-11 through lactylation of NEDD4's K33 site.

### Lactate and lactylation inhibitors reduce APAP-induced liver injury

**Figure 9:**
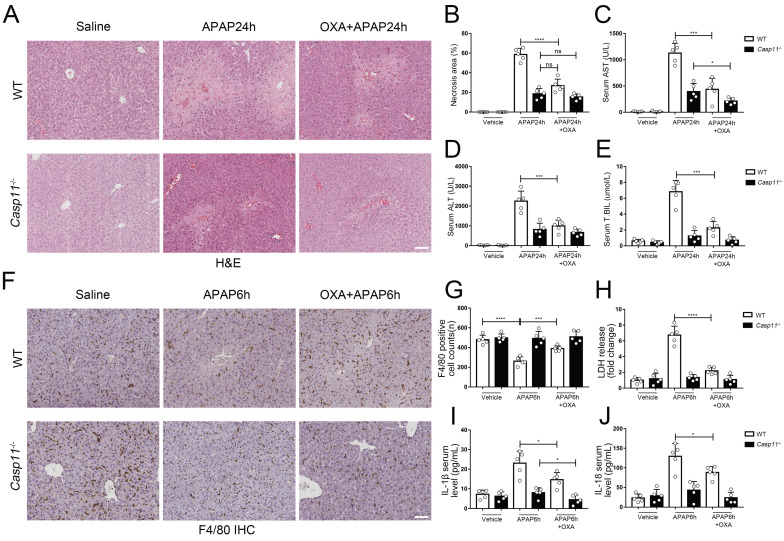
** Lactate inhibitor can reduce APAP-induced liver injury. A** To inhibit lactate production, sodium oxamate (OXA, 0.5 g/kg body weight) was i.p. injected 6 h before APAP treatment. H&E staining of liver tissues from WT and *Casp11*^-/-^ mice, showing necrosis in the liver (images are representative of five independent experiments, scale bars represent 50 μm). **B** Necrotic areas in the liver of WT and *Casp11*^-/-^ mice(n=5). **C-E** Serum levels of AST, ALT, T.BIL in WT and *Casp11*^-/-^ mice after APAP treatment for 24h, measured by ELISA(n=5).** F** F4/80 staining of the liver tissues from WT and *Casp11*^-/-^ mice after 6h of APAP treatment (images are representative of five independent experiments, scale bars represent 50 μm, each group contains 5 mice). **G** Quantification of F4/80 positive cells in each field of the liver tissues from WT and *Casp11*^-/-^ mice showing the survival of macrophages(n=5). **H-J** Serum levels of LDH, IL-1β, IL-18 in WT and *Casp11*^-/-^ mice 6h after APAP treatment, measured by ELISA(n=5). (*p < 0.05, **p<0.01, ***p < 0.001, ****p<0.0001, compared to the other group, n=5 means representative of at least five independent experiments).

Finally, we evaluated the effect of lactylation on AILI in mice models. We administered APAP while suppressing lactate and lactylation levels with oxamate, C646, respectively, and then measured markers of liver injury in both wild-type (WT) and Casp11^-/-^ mice 24 hours later. After using oxamate, the serum lactate levels of APAP intervention mice were significantly reduced (Fig. S8E). We observed a significant improvement in liver damage in WT mice after treatment with oxamate (Fig. 9A) or C646 (Fig. S8A). Additionally, the necrosis area in the liver was reduced (Fig. 9B, Fig. S8B), and the serum markers for liver injury were significantly decreased (Fig. 9C, D, and E). C646 treatment resulted in a reduction of the average necrotic area in *Casp11*^-/-^ mouse livers; however, statistical significance was not achieved., which may be due to its interference with the P300-mediated inflammatory pathway.

To examine the impact of lactate or lactylation on macrophage viability, we assessed the viability of macrophages in both WT and* Casp11*^-/-^ mice 6 hours after inducing AILI. Our results showed that oxamate and C646 significantly reduced macrophage death in WT mice (Fig. 9F, G, Fig. S8 C, D). Additionally, the levels of pyroptosis-related markers, such as LDH, IL-1β, and IL-18, were significantly reduced after treatment with oxamate, but did not return to their baseline levels (Fig. 9H, I, and J). In line with our previous findings, oxamate did not improve macrophage survival or LDH levels in *Casp11*^-/-^ mice and had limited effects on IL-1β and IL-18. Taken together, these results indicate that inhibition of lactylation can alleviate AILI by regulating non-canonical pyroptosis in macrophages.

## Discussion

Our research provides a new insight into this phenomenon by demonstrating that lactate can regulate the function of NEDD4, a crucial negative regulator of Caspase-11, through lactate modification. This leads to the non-canonical pyroptosis of macrophages, which exacerbates the secondary damage in AILI.

It has been well established that Acetaminophen (APAP) disrupts lactate metabolism^51^. Earlier research by Zhang et al. showed that lactate can affect Caspase-8-GSDMC-dependent pyroptosis through upregulation of L-2HG^8^. Our findings indicate that lactate directly promotes Caspase-11-dependent pyroptosis. Previously, there was also evidence that lacate can promote the occurrence of NETosis, which is believed to be GSDMD dependent, that may be an indirect evidence of lactate promoting pyroptosis^12^, ^64^, ^65^. Zhang et al. were the first to discover that lactate can modify histones to regulate gene transcription^13^. Although many non-histone sites of lactylation have been identified, research into their function remains limited. Yang et al. showed that lactylation promotes changes in the cellular localization of HMGB1^15^, while Xiong et al. showed that lactylation enhances METTL3-driven m6A methylation^17^. Our research demonstrates that lactylation can weaken the function of the E3 ubiquitin ligase NEDD4 and highlights the diverse manner in which lactylation can modify protein function, contrasting with reports that acetylation enhances NEDD4's function^22^. Additionally, we observed that NEDD4 lactate levels are regulated by both endogenous and exogenous lactate, and that they are also closely related to the write/erase functions of P300/SIRT1, despite previous reports linking these enzymes with NEDD4 acetylation^22^, ^61^. This suggests that while acetylation and lactylation share the same set of write/erase enzymes, the function of protein lactylation differs from acetylation. Studies on non-canonical inflammasome activation suggest that Caspase-11 may need to interact with NLRP3 for activation, and that this interaction may occur on the dTGN^33^, where NLRP3 is recruited by a negatively charged phosphate-Ptdlns4P^66^. The site of lactylation on NEDD4, which we discovered through LC-MS, is located in the C2 domain. We hypothesize that lactylation at K33 impairs NEDD4 binding to the membrane, reducing its binding to Caspase-11 and leading to its release from ubiquitination. Given that LPS/invading pathogens can also increase lactate levels in macrophages^67^, this may also be a mechanism by which the inhibition of Caspase-11 is lifted during the immune response, allowing the body to quickly eliminate pathogens^68^. This study provides a crucial insight into removing the negative regulation of Caspase-11.

In the past, it was believed that liver cell death in AILI was not due to pyroptosis^69^, but recent evidence suggests otherwise. There is evidence that hepatocytes are resistant to Caspase-1/11-induced pyroptosis^47^. Our research results also showed that the knockout of Caspase-11 did not affect the viability of hepatocytes under APAP treatment. This prompted us to shift the research subject from hepatocytes to macrophages^70^, as their involvement has not yet been fully understood^30^.

Previous research has shown that the number of F4/80^+^ cells, which are macrophages, rapidly decreases in the liver soon after APAP intervention^71^-^73^, which is known as "macrophage disappearance response"^74^. This has been previously believed to be caused by apoptosis of macrophages^75^ but it is difficult to distinguish between apoptosis and pyroptosis. We also observed a rapid reduction in the number of macrophages in early AILI. Since hepatocytes are unlikely to be the source of rapidly elevated markers of pyroptosis in blood and tissues^47^, we believe that the pyroptosis of macrophages mediates this phenomenon. As oxidative stress generated by AILI may be closely related to NLRP3 activation, as reported in our previous study^76^, canonical pyroptosis was inevitably the first to be paid attention to^50^. We discovered that non-canonical pyroptosis can promote secondary injury in AILI through inflammation^42^, and that the knockout of Caspase-11 greatly inhibited the production of serum biomarkers of pyroptosis. This suggested that the non-canonical pathway is more important than the canonical pathway in AILI. As mentioned in our results, the severe inflammation resulting from non-canonical pyroptosis in the early stage of AILI is distinct from the inflammatory response resulting from hepatocyte necrosis in terms of kinetics. This rapid exacerbation of liver damage is evident in the survival status of mice given lethal doses of APAP. Our conclusion does not contradict previous findings that inhibiting NLRP3 can alleviate AILI, as Caspase-11 can activate the NLRP3 inflammasome through a non-canonical, GSDMD-dependent pathway^45^.The neglect of non-canonical pyroptotic pathways in the past may be due to the belief that the core process of AILI is sterile inflammationn^77^. Growing evidence suggested that pathogens colonizing the body affect the course of AILI^78^-^80^. For instance, a study by Su et al. demonstrated that inhibiting LPS binding protein (LBP) could alleviate AILI, and elevated LPS levels were observed in portal blood during the early stages of AILI^26^. Another pre-clinical study by Lee et al. indicated that clearing LPS could alleviate AILI^31^. We also obtained similar results in portal blood, which may be related to impaired hepatic immune barrier following macrophage loss^73^, ^81^. Taken together, LPS may play a role in exacerbating liver damage through a Caspase-11-dependent non-canonical pyroptotic pathway during AILI.

There are some limitations in the current research on lactylation and its relationship with DILI. Firstly, the lack of clinical studies and data on lactylation makes it difficult to obtain direct clinical evidence for the relationship between lactylation and the prognosis of AILI patients. Further investigation through a prospective cohort study may be necessary to clarify this relationship. Secondly, there is no specific agonist or antagonist available to regulate lactylation or the lactylation of a specific protein, and the knockout of NEDD4 leads to a lethal mutation^19^, which makes it challenging to study the effect of NEDD4 lactylation on AILI at the animal level.

In conclusion, this study sheds new light on the role of lactate/lactylation in the non-canonical pyroptosis of macrophages. Our findings indicate that lactate directly contributes to the lactylation of NEDD4 by adding lactyl- groups to the protein, which is dependent on the levels of endogenous and exogenous lactate and the activity of p300/SIRT1. This process leads to the inhibition of NEDD4 binding to Caspase-11, thereby inhibiting the ubiquitination and increasing the protein levels of Caspase-11, resulting in upregulated non-canonical pyroptosis and aggravated secondary damage of AILI. In summary, our research highlights a critical regulatory mechanism for Caspase-11 restriction during non-canonical inflammasome activation, providing a potential target for the treatment of AILI and other diseases related to non-canonical pyroptosis.

## Supplementary Material

Supplementary figures and table.
