# Supplementary material for: NEDD4 lactylation promotes APAP induced liver injury through Caspase11 dependent non-canonical pyroptosis
Source: Int J Biol Sci. 2024 Feb 4;20(4):1413–35. doi: 10.7150/ijbs.91284 (PMC10878146; doi:10.7150/ijbs.91284)
Supplement: Supplementary file 1 — Supplementary figures and table. [file ijbsv20p1413s1.pdf]

Supplemental Information of "NEDD4 lactylation promotes APAP induced liver injury through Caspase11 dependent non-canonical pyroptosis."

Figure and Figure legends

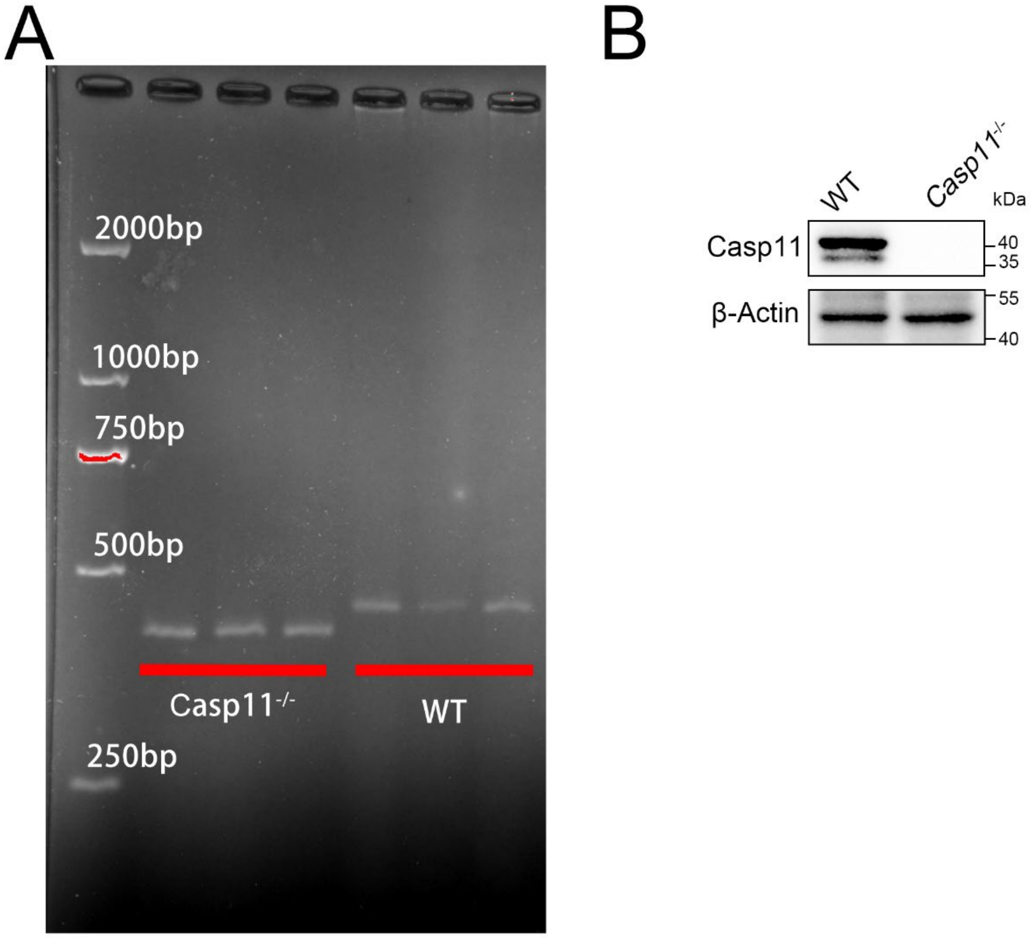

FigS1 A Identification of mice genotype by RT-PCR assay. **B** Identification of mice genotype by Western blotting assay.

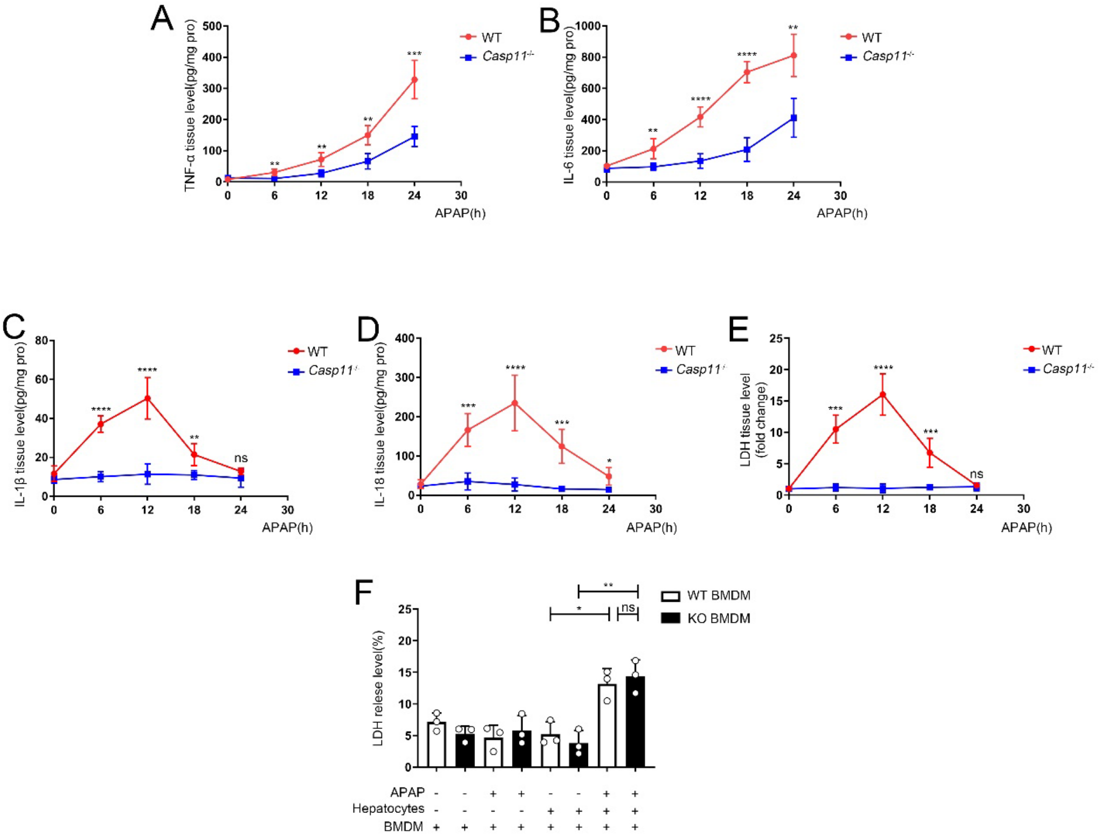

FigS2 A-E Tissue level of TNF-α, IL-6, IL-1β, IL-18, LDH among WT and Casp11$^{-/-}$ mice after treated by APAP 0, 6, 12, 18, 24h were measured by ELISA(n=5). **F** The releasing levels of LDH were detected (n=3). (*p < 0.05, **p<0.01, ***p < 0.001, ****p<0.0001, compared to the other group at the same time).

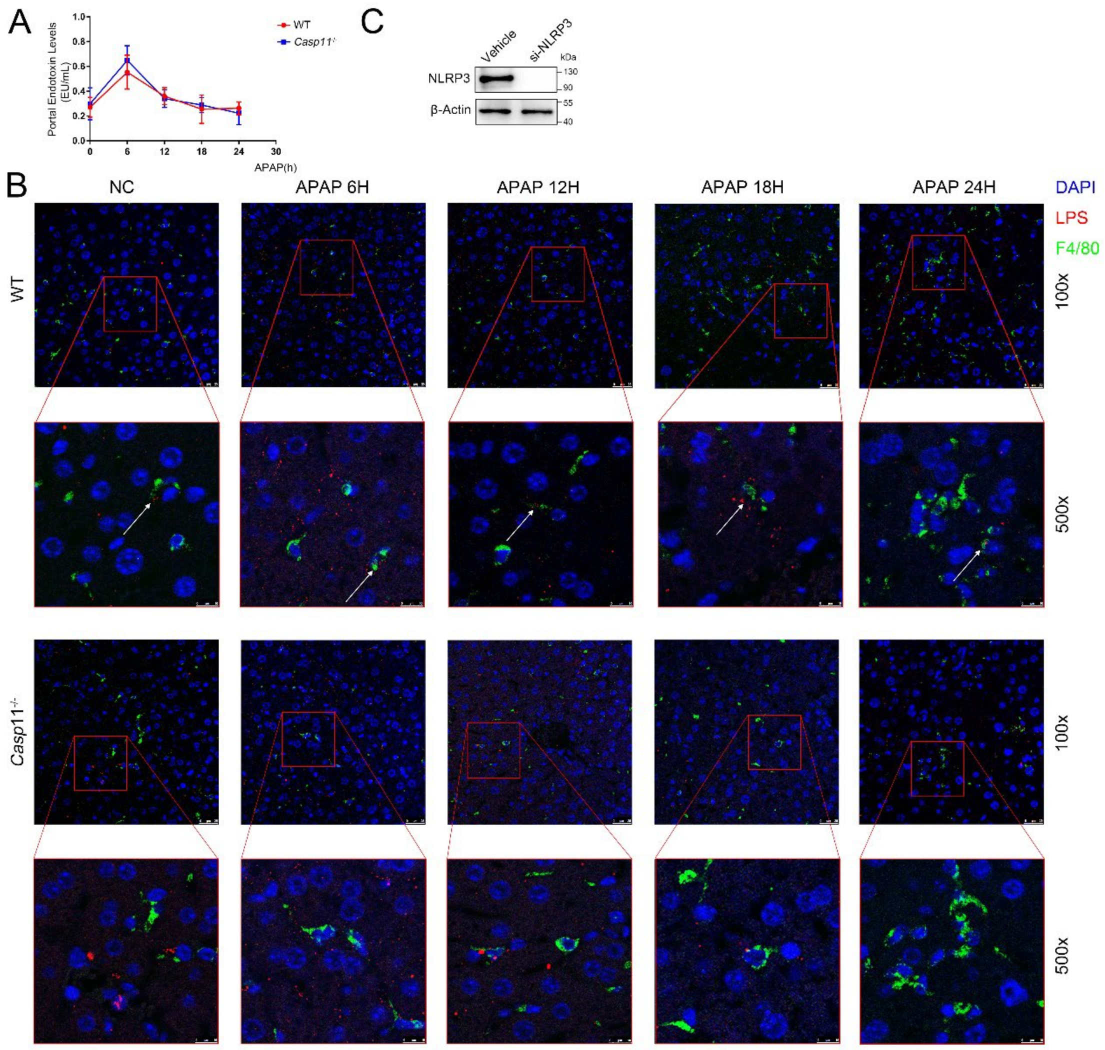

FigS3 **A** Endotoxin levels in the portal vein of WT and Casp11$^{-/-}$ mice after APAP treatment for 0, 6, 12, 18, 24h were measured by LAL assay (n=5). **B** Immunofluorescence staining of liver tissues from WT and Casp11$^{-/-}$ mice showing LPS and F4/80. LPS (red) and F4/80 (green) were examined by confocal microscope. Nuclei were counterstained by DAPI (blue) staining. **C** Identification of si-NLRP3 by Western blotting assay.

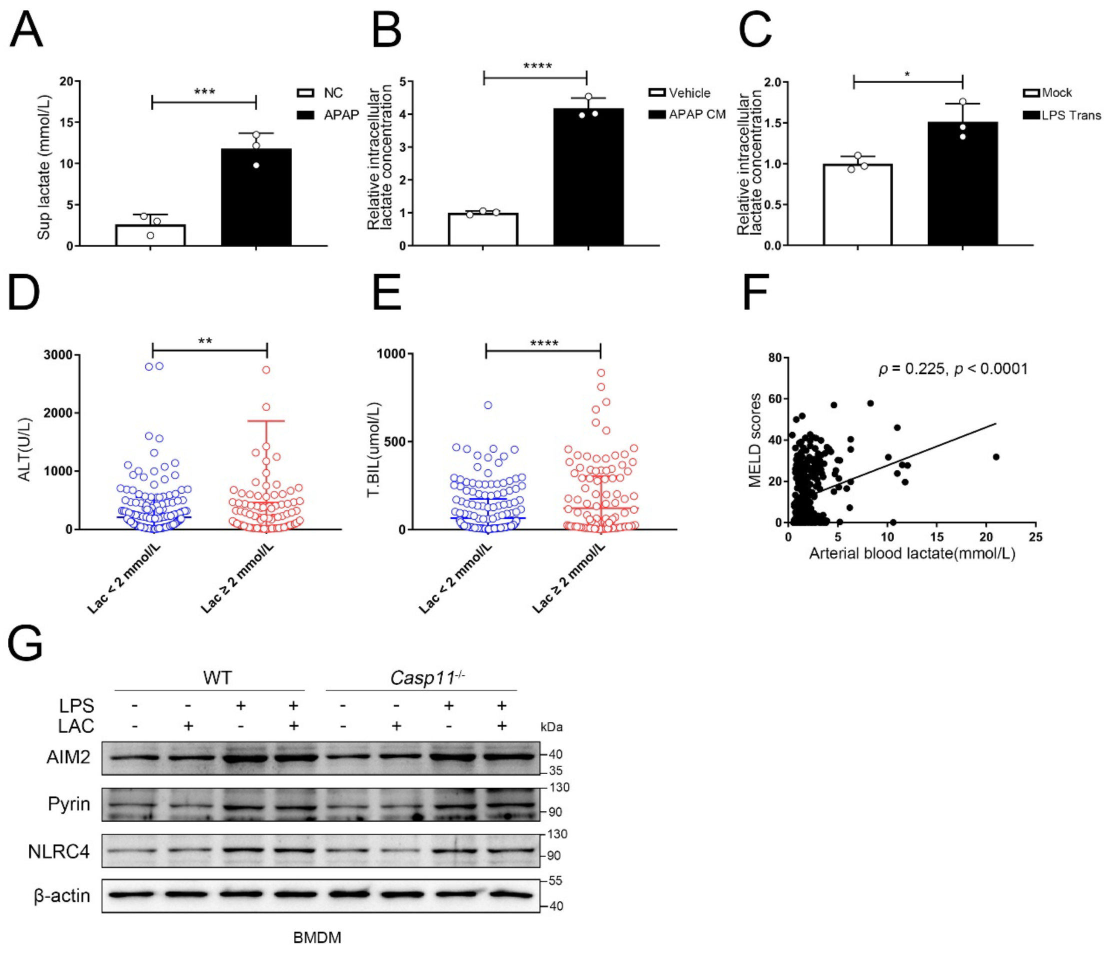

FigS4 **A** Lactate levels of supernatant from hepatocytes treated with APAP. **B-C** Lactate levels of cytoplasm from BMDM treated with APAP CM and LPS transfection. **D-E** Serum ALT and T.BIL levels among DILI patients who were divided into 2 groups according to their lactate levels. **F** Linear regression showing the correlation between MELD score and blood lactate levels of patients. **G** Cell lysate of WT and Casp11$^{-/-}$ BMDMs treated by PBS, LAC (25mM), LPS (500 ng/mL), and LPS+LAC were analyzed by Western blot using antibodies against AIM2, Pyrin, NLRC4 and β-actin(n=3) (*p < 0.05, **p<0.01, ***p < 0.001, ****p<0.0001, compared to the other group at the same time).

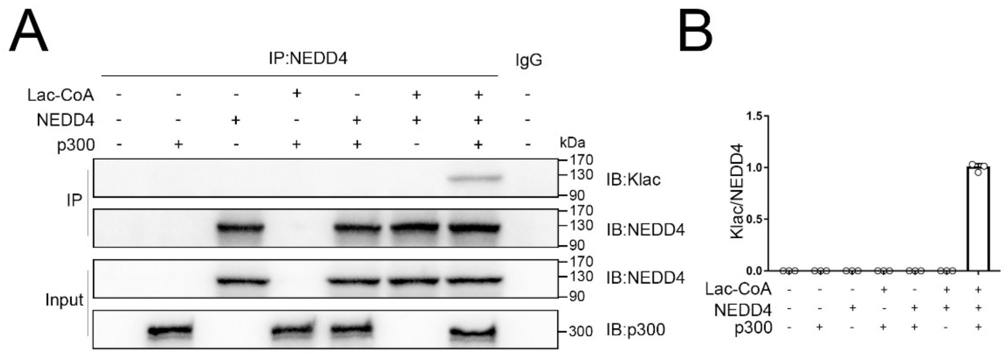

FigS5 P300 catalyzes NEDD4 lactylation in a Lac-CoA dependent manner.

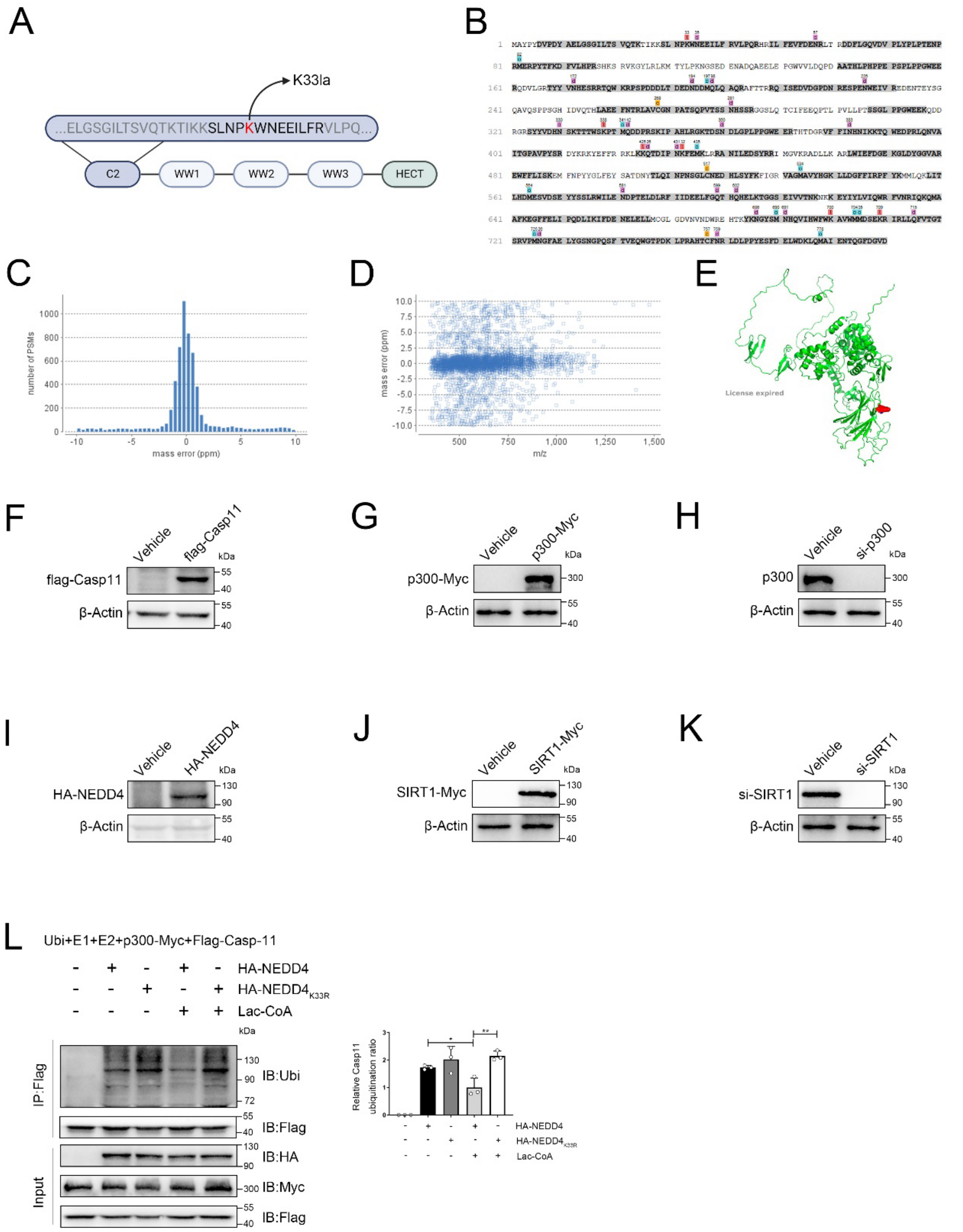

FigS6 A Schematic diagram of molecular structure of NEDD4 K33la. The red position indicates the lactate modification at the K33 site **B** All peptides found in our search from HA-NEDD4 was shown in figure. **C** Precursor mass error of peptide-spectrum

matches (PSM) in filtered result. Distribution of precursor mass error in ppm. **D** Scatterplot of precursor m/z versus precursor mass error in ppm. **E** Schematic diagram of the location of the NEDD4 lactylation site. **F-K** Western blot data confirming knockdown or overexpression of certain targets. **L** In vitro ubiquitination assays were used to measure purified Casp11 that was incubated with P300, HA-NEDD4 WT, HA-NEDD4K33R, which were immunoprecipitated from HEK293T cells and then analyzed via immunoblotting with an anti-Ubi antibody to detect Casp11 ubiquitination.

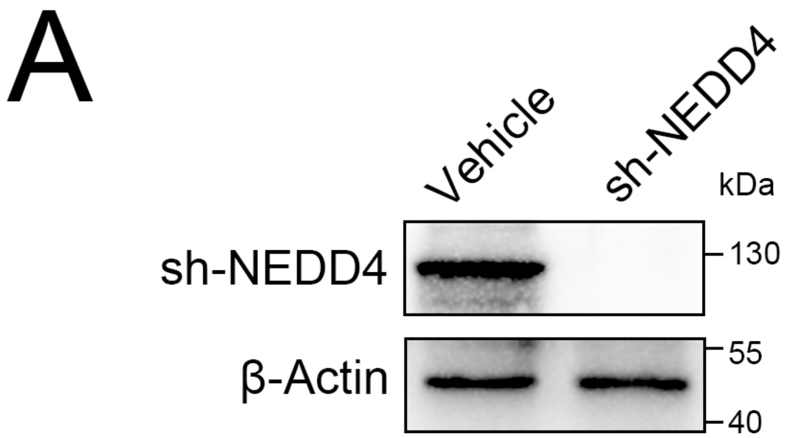

FigS7 A Western blot data confirming knockdown or overexpression of certain targets.

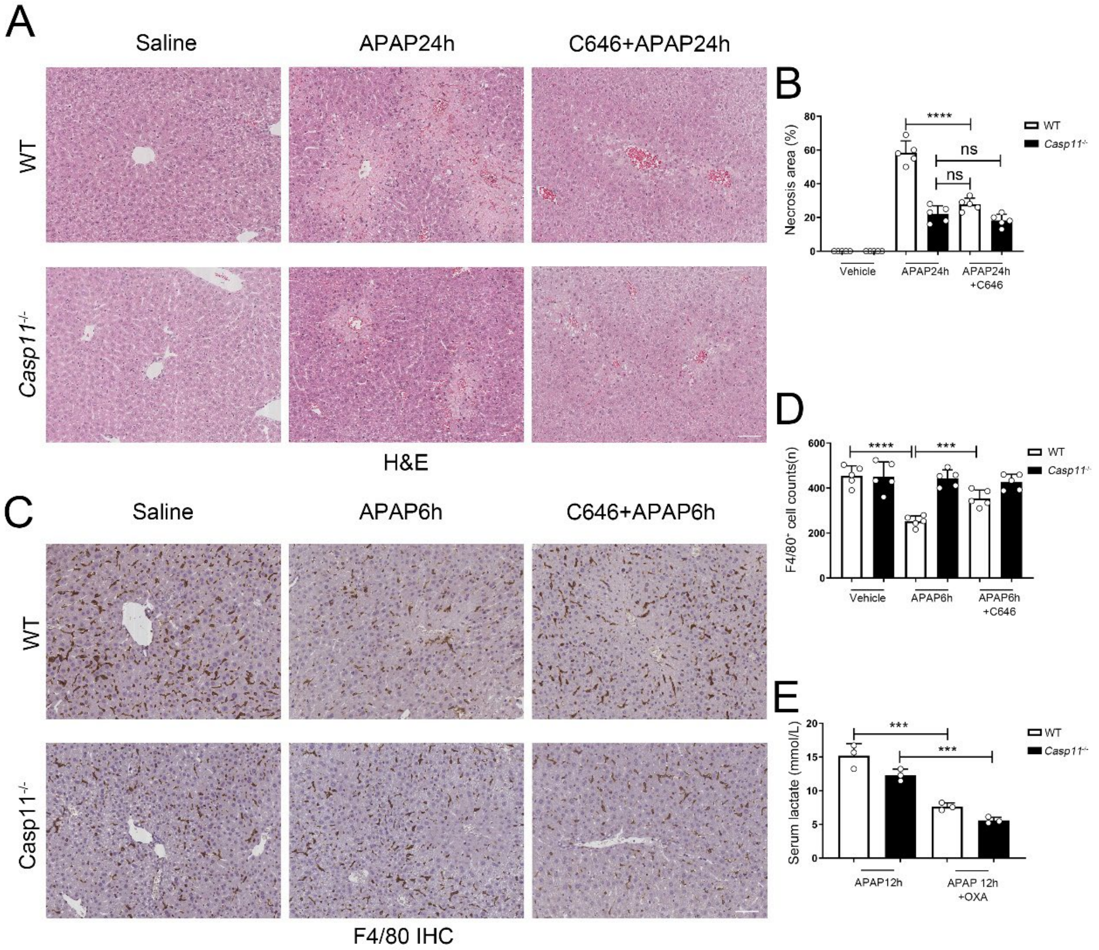

Fig S8 A H&E staining of liver tissues from WT and Casp11$^{-/-}$ mice, showing necrosis in the liver (images are representative of five independent experiments, scale bars represent 50 μm). **B** Necrotic areas in the liver of WT and Casp11$^{-/-}$ mice(n=5). **C** F4/80 staining of the liver tissues from WT and Casp11$^{-/-}$ mice after 6h of APAP treatment (images are representative of five independent experiments, scale bars represent 50 μm, each group contains 5 mice). **D** Quantification of F4/80 positive cells in each field of the liver tissues from WT and Casp11$^{-/-}$ mice showing the survival of macrophages(n=5). **E** Serum lactate levels among WT and Casp11$^{-/-}$ mice after being treated by Oxamate 6h follow APAP 12h were measured by ELISA assay(n=5). (*$p < 0.05$, **$p<0.01$, ***$p < 0.001$, ****$p<0.0001$, compared to the other group, n=5 means representative of at least five independent experiments).

Supplementary Table of “NEDD4 lactylation promotes APAP induced liver injury through Caspase11 dependent non-canonical pyroptosis.”

Table S1

**Reagents**	
	Source
APAP	Sigma
Lactate	Sigma
Sodium lactate	Sigma
Pam3CSK4	Invivogen
LPS	Invivogen
Lipofectamine 3000	ThermoFisher
CCK8 assay Kit	Beyond time Inc.
C646	Sigma
CHC	Sigma
Oxamate	Sigma
EX527	Selleckchem
SRT2183	Selleckchem
MG-132	Sigma

**Antibody**		
	Dilution	Cat. Number
Caspase-11	1:1000 for western blot;	Abcam; ab180673
CYP2E1	1:5000 for western blot;	Abcam; ab28146
JNK	1:1000 for western blot;	CST; 9252
p-JNK	1:1000 for western blot;	CST; 4668
β-actin	1:10000 for western blot;	Abcam; ab6276
F4/80	1:100 for IHC;1:100 for IF	Abcam; ab6640
LPS	1:100 for IF;	Abcam; ab35654
GSDMD	1:1000 for western blot;	Abcam; ab209845
Ubi	1:1000 for western blot;	Abcam; ab134953
NEDD4	1:1000 for western blot; 1:100 for IF	Proteintech; 21698-1-AP
Caspase-11	1:100 for IF	CST; # 14340
Anti-HA tag	1:1000 for western blot; 1:50 for IP	Abcam; ab1424
Anti-flag tag	1:1000 for western blot; 1:100 for IF;1:50 for IP	Sigma; F1804
Anti-Myc tag	1:1000 for western blot; 1:50 for IP	CST; #2278
SIRT1	1:1000 for western blot;	Proteintech; 13161-1-AP
P300	1:1000 for western blot;	Immunoway; YT5693
Pan-kla	1:1000 for western blot;	PTM; PTM-1401-RM

**Small Interfering RNA**		
	Source	Cat.
si-NC	ThermoFisher	Cat# AM4611

si-P300	ThermoFisher	Cat# 4390771 (siRNA ID s116225)
si-CBP	ThermoFisher	Cat# AM16708 (siRNA ID 283827)
si-NLRP3	ThermoFisher	Cas# AM16708(siRNA ID 169997)
